# Comparison of Methods for Sensitivity Analysis of Heterogeneous Treatment Effects in Observational Studies and Application to Alzheimer's Disease and Cognitive Decline

**DOI:** 10.1002/sim.70446

**Published:** 2026-03-17

**Authors:** Jingqi Duan, Corinne D. Engelman, Qiongshi Lu, Hyunseung Kang

**Affiliations:** ^1^ Department of Statistics University of Wisconsin Madison Wisconsin USA; ^2^ Department of Population Health Sciences University of Wisconsin Madison Wisconsin USA; ^3^ Department of Biostatistics and Medical Informatics University of Wisconsin Madison Wisconsin USA

**Keywords:** cognitive decline, heterogeneous treatment effects, optimal matching, sensitivity analysis, sleep

## Abstract

In Alzheimer's disease (AD) research, many observational studies have shown that the effect of sleeping quality, a modifiable risk factor, on cognitive decline is heterogeneous, where some adults experience faster rates of cognitive decline compared to others. However, these effects are likely confounded by unmeasured confounders, and the sensitivity of these effects to unmeasured confounders may be heterogeneous, where one subgroup's treatment effect is more sensitive than that of another subgroup. Unfortunately, compared to the overall treatment effect, there are limited investigations about the sensitivity of heterogeneous treatment effects to unmeasured confounding. The paper presents and compares methods for sensitivity analysis of heterogeneous effects in observational studies based on Rosenbaum's model for sensitivity analysis. We show that, unlike the sensitivity analysis of the overall treatment effect, the sensitivity of heterogeneous treatment effects depends on the variation in the effect sizes across subgroups and the correction for multiple testing. The data analysis further supports our findings where the overall effect of sleep disturbances on cognitive decline is significant (p‐value = $5.55\times 10^{-5}$). Also, the effect is more severe among males (p‐value = $2.00\times 10^{-4}$) and insensitive to a moderate degree of unmeasured confounding. Finally, we offer an easy‐to‐use R software to carry out the sensitivity analyses for heterogeneous treatment effects.

**Abbreviations**
AD: Alzheimer's Disease
APOE: Apolipoprotein E
CART: classification and regression tree

## 1 Introduction

### 1.1 Motivation: Sleep, Effect Heterogeneity, and Unmeasured Confounding

Sleep problem is a well‐known, modifiable risk factor for dementia and Alzheimer's Disease (AD). Numerous observational studies have reported associations between poor sleep quality and cognitive decline [1, 2, 3, 4, 5, 6, 7, 8, 9, 10, 11]; see Yaffe et al. [6] and Ju et al. [12] for comprehensive reviews. Some studies [8, 13] have further suggested that this association is more pronounced in males than in females. However, a major limitation of these findings is that the estimated heterogeneity may be confounded by unmeasured confounders. Specifically, even after adjusting for known confounders of cognitive decline, such as family history of dementia [14, 15] and possession of the Apolipoprotein E ε4 (APOE4) allele [16, 17, 18, 19], many environmental factors, including lifestyle, occupation, diet and exercise, are often unmeasured and may confound the relationship between sleep problem and cognitive decline [6].

More broadly, when studying heterogeneous treatment effects in observational studies, unmeasured confounders may influence effect heterogeneity, as they can moderate the relationship between treatment and outcome in much the same way as measured confounders. If unmeasured confounders were observed and appropriately adjusted for, the estimated treatment effect for a subgroup based only on measured confounders could differ substantially. Importantly, the biases introduced by unmeasured confounders can differ across subgroups, leading to nuanced downstream consequences that are more complex than those encountered when estimating the overall treatment effect.

As a concrete example of some of these nuanced consequences, consider our motivating example again, where the effect of sleep problem (treatment) on cognitive decline (outcome) has been observed to be larger among males than females. Psychological stress is an important environmental confounder associated with both sleep problem and cognitive decline. When it is unmeasured, it may not only bias the estimated treatment effects, but also alter conclusions about effect heterogeneity by sex. For example, if the variation in stress is similar among males and females, then adjusting for it is unlikely to change the conclusion about effect heterogeneity; the estimated effects would shift similarly, and the effect in males would remain larger. However, if the variation in stress is greater in males, or in the extreme case, nearly constant in females, the estimated effect among females would be approximately unbiased, as little to no variation in stress restricts its potential to confound the relationship. In contrast, greater variability of stress in males may introduce more confounding bias, meaning that adjusting for stress could substantially change the estimated effect, such that the treatment effect in males may no longer be larger than that in females.

### 1.2 Related Works and Our Contribution

Sensitivity analysis is a popular method to address the impact of unmeasured confounding in observational studies [20, 21, 22, 23, 24, 25, 26]. Most of these approaches focus on sensitivity analysis for the overall or average treatment effect, where the estimand of interest is a single scalar quantity. Importantly, the estimand is fixed in advance rather than being selected in a data‐driven manner. Two notable exceptions are the sensitivity analyses proposed by Hsu et al. [27], referred to as HSR, and by Lee et al. [28], referred to as submax. Both HSR and submax extend sensitivity analysis to settings with heterogeneous treatment effects, where the estimand of interest is typically a vector, for example, the average treatment effect within each subgroup. HSR uses matched pairs to construct a classification and regression tree (CART) for identifying subgroups and conducts sensitivity analysis within each subgroup. In contrast, submax generalizes beyond matched pairs to accommodate matched sets with multiple controls and uses the “subgroup maximum method” (i.e., submax) to perform sensitivity analysis across predefined subgroups. Although submax primarily focuses on settings where the subgroups are defined a priori, Section 6 of Lee et al. [28] outlines how the method can be extended to data‐driven subgroup identification. Both methods use Rosenbaum's model for sensitivity analysis [22] and do not require sample splitting, which typically divides the original sample into two subsets: one for subgroup identification and the other for treatment effect estimation. Moreover, both methods have been shown to control the familywise error rate; that is, the probability of rejecting at least one true null hypothesis does not exceed the specified significance level.

The primary contribution of the paper is a comparative evaluation of two existing methods and a hybrid approach, referred to as submax+ (see Section 3.2 for details) with respect to (a) statistical power and (b) the ability to detect true effect modifiers, and to provide guidance for selecting methods for sensitivity analysis in practice. A discussion by Lee and Hsu [29] noted that HSR, submax, and submax+ do not uniformly dominate one another. Our specific aim is to identify settings where submax performs uniformly better than HSR or submax+ (or vice versa), as well as settings where the three methods exhibit mixed performance. We restrict our comparison to these three approaches because they all rely on the same underlying model for sensitivity analysis and, to our knowledge, no existing method for sensitivity analysis of heterogeneous treatment effects permits subgroup discovery, treatment effect estimation, and sensitivity analysis to be conducted on the same sample (i.e., same n) without inflating the Type I or familywise error rate.

As part of our comparison, we clarify several implementation details of the existing methods that we believe will be helpful to practitioners and introduce a few modest improvements. For example, we provide a detailed discussion on how to modify matched sets after subgroups have been identified by CART, with the goal of increasing the number of matched sets while preserving good covariate balance. In addition, we incorporate pair matching and variable‐ratio matching with almost exact matching and near‐fine balance [22, 30, 31] to improve covariate balance. We also perform amplification analysis [32] to enhance the interpretability of Rosenbaum's sensitivity parameter Γ≥1.

We also compare the methods by replicating the analysis of the effect of sleep problems on cognitive decline using data from the Alzheimer's disease neuroimaging initiative (ADNI). ADNI is a longitudinal study launched in 2003 to investigate mild cognitive impairment (MCI) and early AD; see Section 2.1 for details on the study population, exposure, and outcome. Consistent with prior research, we find that the overall treatment effect of sleep disturbances is significant (p‐value = $5.55\times 10^{-5}$), and we discover that the effect is most pronounced among males (p‐value = $2.00\times 10^{-4}$). More importantly, and in contrast to previous studies, we quantify the potential impact of unmeasured confounding on the estimated effects within each subgroup. Specifically, the estimated effect among males is insensitive to a moderate level of unmeasured confounding (Rosenbaum's Γ=1.8), while the effect among APOE4 carriers is insensitive to a slightly lower level of unmeasured confounding (Rosenbaum's Γ=1.6).

Finally, we provide an easy‐to‐use R package, sensitivityhte, that implements subgroup identification and sensitivity analyses of HSR and submax+ . While several R packages exist for estimating heterogeneous treatment effects (e.g., grf [33], bartCause [34], and DoubleML [35]), to our knowledge, no existing package supports both the estimation of heterogeneous treatment effects and sensitivity analysis for unmeasured confounding. Moreover, neither HSR nor submax+ has previously been implemented in an R package; submax is available via the R package submax [36]. Our package enables users to adaptively identify subgroups based on their matching results and perform sensitivity analysis within each subgroup. We hope the package makes these methods more accessible to applied researchers and facilitates the application of sensitivity analysis for heterogeneous treatment effects in observational studies.

## 2 Setup

### 2.1 Data Setup

This section provides a summary of our dataset, which we use to compare the methods. The ADNI was launched in 2003 as a public‐private partnership, led by Principal Investigator Michael W. Weiner, MD. The primary goal of ADNI has been to test whether serial magnetic resonance imaging (MRI), positron emission tomography (PET), other biological markers, and clinical and neuropsychological assessments can be combined to measure the progression of MCI and early AD. For up‐to‐date information, see http://www.adni‐info.org. Data used in the preparation of this article were obtained from the ADNI database (http://adni.loni.usc.edu), collected from adults aged between 55 and 90 at 57 sites in the United States and Canada.

A total of 2430 individuals were recruited during the ADNI1, ADNIGO, and ADNI2 phases. We excluded individuals from our analysis if: (1) the presence of sleep problems (see next paragraph for the exact definition) at the baseline visit could not be determined; (2) composite executive function scores were missing either at the baseline visit or at the 24‐month follow‐up visit; or (3) genetic data were unavailable. After these exclusions, 1143 individuals remained. Of them, 1131 individuals (98.95% of 1143 individuals) self‐reported as white in response to a question about race. Given that the ADNI cohort is predominantly white, with 91.93% of all 2430 individuals self‐identifying as white, we restricted our analysis to self‐identified white individuals. Thus, the final sample size for analysis was 1131.

An individual was defined to have sleep problems if they had insomnia or showed abnormal nighttime behaviors, as reported by themselves or their caregiver at the baseline visit. This led to 295 individuals with sleep problems (i.e., the treated group) and 836 individuals without sleep problems (i.e., the control group) at the baseline visit. Among the 295 individuals with sleep problems, 190 individuals had insomnia, 176 individuals had abnormal nighttime behaviors, and 71 individuals had both insomnia and abnormal nighttime behaviors. We remark that alternative definitions of sleep problems have been used in the literature; a detailed review is provided in Appendix Section B.

The outcome was defined as the decline in composite executive function (EF) scores over two years, calculated by subtracting the EF score at the second annual visit from the baseline score. Thus, a positive outcome value indicates a decline in EF (i.e., lower score at follow‐up), while a negative value indicates an improvement. The outcome ranged from −2.539 to 2.324, with a mean of 0.102 and a standard deviation of 0.691. The EF scores were derived by Crane et al. [37] using the ADNI neuropsychological assessment battery.

We controlled for individuals' demographics, including sex, Latino ethnicity, age, retirement age, years of education, handedness, residence type, and marital status. These variables were measured either at the baseline visit or during the screening interview prior to the baseline visit. We also controlled for family history of dementia and AD, specifically the individual's self‐reported parental and sibling history of dementia and AD, as well as the individual's baseline composite memory and EF scores. To adjust for potential genetic confounding, we included polygenic risk scores (PRS) for insomnia, chronotype, and sleep duration as covariates; see Appendix Section C for details. We also adjusted for the number of ε2 and ε4 alleles of APOE, which ranged from 0 to 2 copies. In total, we controlled for 31 covariates, and Table 1 provides a summary of them.

**TABLE 1:** Summary statistics of 31 covariates at baseline.

Covariates	With sleep problems		No sleep problems	
	(*n* = 295)		(*n* = 836)	
	Mean (SD)	%Missing	Mean (SD)	%Missing
Sex (Female)	47.8%		42.3%	
Hispanic	1.0%		1.4%	
Age at baseline, years	73.33 (7.37)		73.81 (6.93)	
Retirement age, years	73.32 (24.03)	2.71%	74.37 (23.87)	1.08%
Education, years	16.06 (2.68)		16.08 (2.79)	
Right‐handed	90.5%		91.7%	
Residence type				
House	72.9%		78.8%	
Condo/co‐op	15.9%		11.0%	
Rented apartment	4.4%		5.1%	
Retirement community	3.4%		2.9%	
Mobile home	1.7%		1.0%	
Assisted living	0.0%		0.6%	
Marital status				
Married	75.6%		78.3%	
Widowed	13.2%		10.0%	
Divorced	6.1%		8.7%	
Never married	4.1%		2.8%	
Family history				
Mom with dementia	42.9%	2.0%	39.8%	2.4%
Mom with AD	28.3%	10.2%	26.4%	8.1%
Dad with dementia	20.9%	6.1%	17.6%	4.1%
Dad with AD	12.3%	9.2%	9.3%	7.5%
Number of female siblings	1.16 (1.24)		1.17 (1.29)	0.84%
Number of siblings w/o dementia and AD	1.94 (1.77)		2.08 (2.06)	0.84%
Number of siblings with dementia	0.07 (0.29)		0.05 (0.27)	0.84%
Number of siblings with AD	0.15 (0.46)		0.14 (0.47)	0.84%
APOE ε2 copies (0/1/2)	90.5/9.5/0.0%		90.4/9.6/0.0%	
APOE ε4 copies (0/1/2)	55.9/35.3/8.8%		53.8/36.4/9.8%	
Polygenic risk scores ($\times 10^{-6}$)				
Insomnia	−6.10 (20.24)		−6.07 (19.93)	
Sleep duration	85.14 (8.64)		84.39 (8.38)	
Chronotype	−38.86 (8.89)		−40.22 (8.21)	
Baseline composite memory score	0.36 (0.89)		0.33 (0.85)	
Baseline composite executive function score	0.30 (0.99)		0.31 (1.00)	

For covariates with missing values, we used a two‐step imputation procedure outlined in Chapter 9.4 of Rosenbaum [22]. For each covariate with missing entries, we (i) created an indicator variable that takes the value 1 for the missing entries and 0 for the non‐missing entries, and (ii) imputed the missing entries in the covariate with the mean of the non‐missing entries. Matching on the covariates and the derived indicator variables balances both the observed covariates and the pattern of missingness, for example, the proportion of missing values for each covariate. But, the missing values themselves may or may not be balanced; see Chapter 13.4 of Rosenbaum [22] for a detailed discussion and an example.

Before adjusting for baseline covariates, we found that the treated group (i.e., individuals with sleep problems) was slightly more likely to have a parental history of dementia or AD compared to the control group (i.e., individuals without sleep problems). The treated group was also more likely to be widowed and to reside in a condo or co‐op. We will adjust for these baseline covariates using optimal matching.

### 2.2 Review: Optimal Matching for Covariate Adjustment

We briefly review optimal matching, which was used to adjust for baseline covariates in the HSR, submax, and submax+ methods. Matching is a popular and interpretable method to adjust for measured covariates. An optimal matching algorithm is a type of matching that matches treated individuals (i.e., individuals with sleep problems) to controls (i.e., individuals without sleep problems) by minimizing the sum of total covariate distances between individuals. In this paper, covariate distance between two individuals was measured using the rank‐based Mahalanobis distance, and a propensity score caliper was used to penalize large distances; see Chapter 8 of Rosenbaum [22] for details.

We also enforced two constraints on the optimal matching algorithm: near‐fine balance and almost exact matching. Near‐fine balance forces the marginal distributions of discrete covariates between the treated and control groups to be nearly identical [22, 30]. Almost exact matching ensures that for a prespecified set of covariates, the matches on these covariates are as exact as possible (see Chapter 9.2 of Rosenbaum [22]). Almost exact matching results in an exact match whenever it is feasible. For software, we used the optmatch R package [38] and the bigmatch R package [31].

Formally, let I denote the number of matched sets, and let the matched sets be indexed by i=1,…,I. For each matched set i, there are $n_{i}$ individuals, indexed by $j=1,\ldots ,n_{i}$, and one of the $n_{i}$ individuals is treated (i.e., an individual with sleep problems), while the remaining $n_{i}-1$ individuals are not treated (i.e., individuals without sleep problems). We remark that if one individual with sleep problems is matched to exactly one individual without sleep problems, that is, $n_{i}=2$, the resulting matching procedure is referred to as optimal pair matching. If one individual with sleep problems is matched to at least one individual without sleep problems, that is, $n_{i}\geq 2$, the resulting matching procedure is referred to as optimal variable‐ratio matching.

### 2.3 Review: Potential Outcomes, Observed Outcomes, and Sharp Null of No Causal Effect

For each individual ij, we denote the potential outcome under treatment as $r_{Tij}$ and the potential outcome under control as $r_{Cij}$. We use $R_{ij}$ to denote the observed outcome and $x_{ij}$ to denote the observed confounders for individual ij. Let $Z_{ij}$ be the treatment indicator, where $Z_{ij}=1$ indicates that the individual was treated and $Z_{ij}=0$ indicates that the individual was under control. The observed outcome is related to the potential outcomes by the equation $R_{ij}=Z_{ij}r_{Tij}+(1-Z_{ij})r_{Cij}$, and we assume that the stable unit treatment value assumption (SUTVA) [39] holds.

For hypothesis testing, we consider Fisher's sharp null hypothesis [40] of no treatment effect, which asserts that $r_{Tij}=r_{Cij}$ for all ij, that is, $H_{0}:r_{Tij}=r_{Cij}$, ∀ij. We can also define the sharp null hypothesis for subgroups of individuals with identical measured confounders. Specifically, suppose we divide the I matched sets into G mutually exclusive and exhaustive subgroups. Let $𝒢=\{s_{1},\ldots ,s_{G}\}$ be the “set of sets”, where each $s_{g}\subseteq \{1,\ldots ,I\}$ denotes a nonempty subgroup of individuals and $s_{g^{\prime }}\cap s_{g}=\emptyset$ for any $g\neq g^{\prime }$. The size of the subgroup $s_{g}$ is denoted as $I_{g}=|s_{g}|$ and $\sum_{g=1}^{G}I_{g}=I$. The sharp null hypothesis for subgroup $s_{g}$ asserts no treatment effect for all matched sets in $s_{g}$, that is, $H_{g}:r_{Tij}=r_{Cij}$ for all $i\in s_{g}$ and $j=1,\ldots ,n_{i}$.

Given a significance level α∈(0,1), we test the sharp null hypothesis for each subgroup $s_{g}$ by assessing whether the weighted M‐statistic $T_{g}$ or its appropriately studentized version exceeds the critical value. Briefly, the weighted M‐statistic is an extension of Maritz's version [41] of Huber's M‐statistic [42], and was proposed by Rosenbaum [43, 44, 45] to increase the power of sensitivity analysis. In our paper, $T_{g}$ is defined as

$$\begin{array}{ll} T_{g} & =\underset{i\in s_{g}}{\sum }w_{i}\overset{n_{i}}{\underset{j=1}{\sum }}Z_{ij}\overset{n_{i}}{\underset{l=1}{\sum }}\psi_{\text{in}}\{(R_{ij}-R_{il})/s\}\\ & \;=\underset{i\in s_{g}}{\sum }w_{i}\overset{n_{i}}{\underset{j=1}{\sum }}Z_{ij}q_{ij},\;\;q_{ij}=\overset{n_{i}}{\underset{l=1}{\sum }}\psi_{\text{in}}\{(R_{ij}-R_{il})/s\}. \end{array}$$

Each $w_{i}$ represents nonnegative weights for matched set i in subgroup g and $\psi_{\text{in}}$ is a monotone increasing odd function that emphasizes certain values of the outcome. For instance, with $\psi_{\text{in}}(y)=y$ and constant weights $w=s{\left(\sum_{i\in s_{g}}n_{i}-1\right)}^{-1}$, $T_{g}$ yields the permutational *t*‐test. Note that $q_{ij}$'s are functions of the $R_{ij}$ and $R_{ij}=r_{Cij}$ under $H_{0}$. For further details, see Appendix Section A.

For multiple testing, we use the closed testing procedure by Marcus et al. [46], which is guaranteed to control for the familywise error rate in the strong sense. Briefly, let 𝒦⊆{1,…,G} be a nonempty subset of the subgroups of matched sets. Define $H_{𝒦}$ as the hypothesis of no treatment effects in the subgroups defined by 𝒦, that is, $H_{𝒦}:r_{Tij}=r_{Cij}$ for all $i\in s_{k}$ and for all k∈𝒦. The closed testing procedure rejects $H_{𝒦}$ at level α if for every set ℒ where 𝒦⊆ℒ⊆{1,…,G}, $H_{ℒ}$ is rejected at level α. Marcus et al. [46] showed that the probability that the closed testing procedure falsely rejects at least one true $H_{𝒦}$ is at most α.

### 2.4 Review: Rosenbaum's Model for Sensitivity Analysis

We briefly summarize Rosenbaum's model for sensitivity analysis under matching; see Rosenbaum [22] for a textbook introduction. For each matched set i, suppose two individuals, ij and $ij^{\prime }$, with the same observed covariates, $x_{ij}=x_{ij^{\prime }}$, differ in their odds of treatment by at most a factor of Γ≥1. Formally, let $u_{ij}$ denote the unmeasured confounder of individual ij and define the set $ℱ=\{(r_{Tij},r_{Cij},x_{ij},u_{ij}),i=1,\ldots ,I,j=1,\ldots n_{i}\}$. Suppose the treatment assignment probability satisfies the following equation

$$\begin{array}{l} \frac{1}{\Gamma }\leq \frac{\mathrm{Pr}(Z_{ij}=1|ℱ)/\mathrm{Pr}(Z_{ij}=0|ℱ)}{\mathrm{Pr}(Z_{ij^{\prime }}=1|ℱ)/\mathrm{Pr}(Z_{ij^{\prime }}=0|ℱ)}\leq \Gamma \;\text{whenever}\;x_{ij}=x_{ij^{\prime }}. \end{array}$$

A value of Γ=1 indicates that there is no unmeasured confounding, while Γ>1 indicates an unmeasured confounder that biases the treatment assignment by at most Γ. Then, for each Γ≥1, we compute the largest, “worst‐case” distribution of the statistic $T_{g}$, g=1,…,G, under the sharp null hypothesis of no effect, that is,

$$\underset{0\leq u_{ij}\leq 1\forall ij}{\max}\mathrm{Pr}\left(T_{g}\geq t\;|\;ℱ,\overset{n_{i}}{\underset{j=1}{\sum }}Z_{ij}=1,\;\forall i\in s_{g},H_{g}\right),\;t\in \mathbb{R}.$$

The R package sensitivitymw [44] computes an asymptotic approximation of the above expression and the corresponding p‐value for a family of weighted M‐statistics. Typically, we aim to identify the smallest value of Γ that retains the null hypothesis $H_{g}$ at the prespecified significance level α∈(0,1); this is referred to as the sensitivity value [47]. A high sensitivity value indicates that the estimated effect is insensitive to biases from an unmeasured confounder.

To better interpret Γ, Rosenbaum and Silber [32] proposed to “amplify” Γ by mapping it to a set of values (Λ,Δ) such that Γ=(ΛΔ+1)/(Λ+Δ). The parameter Λ measures the relationship between an unmeasured covariate $u_{ij}$ and the treatment assignment $Z_{ij}$ while Δ measures the relationship between an unmeasured covariate $u_{ij}$ and the potential outcome $r_{Cij}$. With the new interpretation, Γ=1.25 can be reinterpreted as an unmeasured confounder that doubles the odds of getting treated (i.e., Λ=2) and doubles the odds of increasing the outcome (i.e., Δ=2).

## 3 Methods for Sensitivity Analysis of Heterogeneous Treatment Effects

### 3.1 Hsu et al.'s Method (HSR)

**FIGURE 1:**
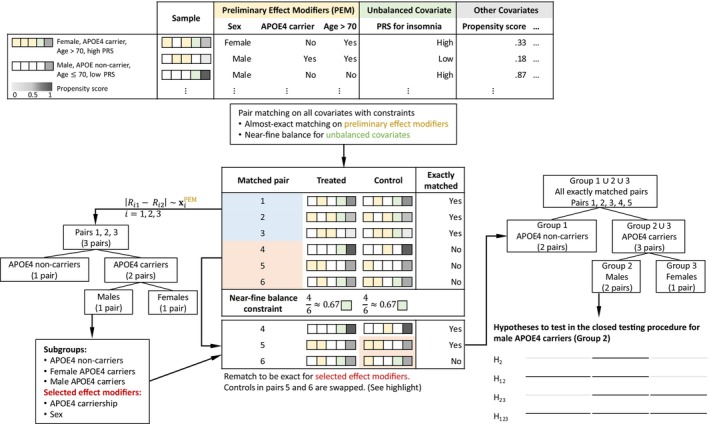
Consider a simple example with sex, APOE4 carriership, and age as preliminary effect modifiers. Optimal pair matching with almost exact matching on these three covariates and near‐fine balance for unbalanced covariates yields I=6 pairs. Pairs 1, 2, and 3 are exactly matched on all preliminary effect modifiers and are used to construct a regression tree by regressing $\left|R_{i1}-R_{i2}\right|$ on $x^{\text{PEM}}$. The tree identifies three subgroups and selects two covariates‐sex and APOE4 carriership‐as effect modifiers. Pairs 4, 5, and 6 are then rematched to be exactly matched on the selected covariates, resulting in two additional pairs exactly matched on sex and APOE4 carriership. $H_{1}$ denotes the hypothesis of no treatment effect for APOE noncarriers (Group 1); $H_{2}$ denotes the hypothesis of no treatment effect for male APOE4 carriers (Group 2); $H_{12}$ denotes the hypothesis of no treatment effect among all APOE4 noncarriers and male APOE4 carriers (Groups 1 and 2); $H_{23}$ denotes the hypothesis of no treatment effect among all APOE4 carriers (Groups 2 and 3); and $H_{123}$ denote the hypothesis of no treatment effect among all groups (Groups 1,2, and 3). The closed testing procedures rejects the hypothesis $H_{2}$ at level α if all four intersection hypotheses involving $H_{2}$ ($H_{2}$, $H_{12}$, $H_{23}$, and $H_{123}$) are rejected at level α.

In this section, we review the method of Hsu et al. [27] (referred to as HSR) for sensitivity analysis of heterogeneous treatment effects; see Figure 1 for a visualization of the procedure. The method begins by selecting a preliminary subset of covariates as potential effect modifiers, denoted $x^{\text{PEM}}$, based on prior knowledge of the treatment effect. This subset may be small or large; in the absence of strong prior knowledge, $x^{\text{PEM}}$ may include all observed covariates. Notably, $x^{\text{PEM}}$ does not necessarily include the “true” effect modifiers.

We perform optimal pair matching with almost exact matching on $x^{\text{PEM}}$ and near‐fine balance constraints. For each pair i exactly matched on $x^{\text{PEM}}$, the treated‐minus‐control difference in observed responses is computed as $Y_{i}=(Z_{i1}-Z_{i2})(R_{i1}-R_{i2})$. Using these exactly matched pairs, a CART [48] is constructed by regressing the absolute differences $\left|Y_{i}\right|$ on $x_{i}^{\text{PEM}}$, where $x_{i}^{\text{PEM}}=x_{i1}^{\text{PEM}}=x_{i2}^{\text{PEM}}$. The CART model is fitted using the rpart R package [49], partitioning $x^{\text{PEM}}$ into subgroups defined by the tree's leaves. Next, we rematch inexactly matched pairs to be exactly matched within the leaf‐defined subgroups, aiming to increase the number of pairs eligible for subsequent analysis. Conducted within the initially matched sample and restricted to inexactly matched pairs, this procedure preserves covariate balance while increasing the number of testable pairs, particularly when CART selects a small subset of covariates from a large pool. For example, exact matching on six binary covariates yields 64 subgroups, often with few matched pairs per subgroup. If CART selects only three covariates, defining four subgroups, rematching on these can recover additional pairs for subgroup hypothesis testing. But, if CART was unable to select all of the true effect modifiers, rematching would make it impossible to test on the covariates that were not selected by CART.

Let G denote the number of leaves in the CART tree. Within each subgroup g=1,…,G, we compute the statistic $T_{g}$ and corresponding p‐value $P_{g}$ to test the sharp null hypothesis of no treatment effect for all matched pairs i in subgroup $s_{g}$, denoted as $H_{g}$. We also test the global null hypothesis, defined as the intersection of all subgroup hypotheses: $H_{\wedge }=H_{1}\wedge H_{2}\wedge \cdots \wedge H_{G}$. That is, $H_{\wedge }$ asserts no treatment effect in any of the G subgroups. The p‐value for testing $H_{\wedge }$ is computed using the truncated product method of combining p‐values [50], defined as $P=\prod_{g=1}^{G}P_{g}^{\chi (P_{g}\leq \tilde{\alpha })}$, where χ(E)=1 if event E occurs and χ(E)=0 otherwise, and the truncation threshold is set to $\tilde{\alpha }=0.05$; see Hsu et al. [27] for details. If $H_{\wedge }$ is rejected, we apply the closed testing procedure [46] described in Section 2.3 to identify the subgroup(s) for which the sharp null is rejected. This procedure can be repeated for different values of Γ≥1 to assess the sensitivity of the conclusion to unmeasured confounding within each subgroup.

### 3.2 Lee et al.'s Method (Submax and Submax+)

**FIGURE 2:**
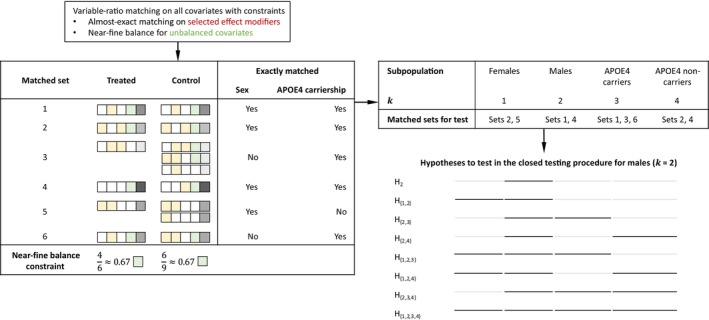
Optimal variable‐ratio matching with almost exact matching on sex and APOE4 carriership and near‐fine balance for unbalanced covariates was applied to all individuals. The submax method allows inclusion of matched sets that are not exactly matched on all selected effect modifiers. For example, matched set 5 is exactly matched on sex but not on APOE4 carriership; thus, it is included in the comparison for females but excluded from comparisons involving APOE4 carriers and noncarriers. For Γ>1, $H_{2}$ refers to the hypothesis of no treatment effect for males (k=2), and $H_{\left\{2,3\right\}}$ refers to no effect for males (k=2) and APOE4 carriers (k=3), allowing for unmeasured confounding up to Γ. The closed testing procedure rejects $H_{2}$ at level α if all eight intersection hypotheses involving $H_{2}$ ($H_{2}$, $H_{\left\{1,2\right\}}$, $H_{\left\{2,3\right\}}$, $H_{\left\{2,4\right\}}$, $H_{\left\{1,2,3\right\}}$, $H_{\left\{1,2,4\right\}}$, $H_{\left\{2,3,4\right\}}$, and $H_{\left\{1,2,3,4\right\}}$) are rejected at level α.

In this section, we review the method of Lee et al. [28] (referred to as submax) and its extension incorporating CART (referred to as submax+ ); see Figure 2 for a visualization of the procedure. The method begins by specifying L binary covariates as potential effect modifiers, with the selection strategy distinguishing submax and submax+ . When covariates are selected based on prior knowledge or include the full set of observed covariates, the procedure is referred to as submax. In contrast, when covariates correspond to the splitting variables identified by the CART tree, as described in Section 3.1, the procedure is referred to as submax+ . We then perform optimal variable‐ratio matching with almost exact matching on the L effect modifiers and near‐fine balance constraints.

We note that submax and submax+ can accommodate both pair matching and variable‐ratio matching, whereas HSR requires pair matching to construct the regression tree. Variable‐ratio matching matches each treated unit with at least one control and allows for flexible matching ratios; for example, a treated individual may be matched to one, two, or more controls. While tempting, we chose not to restrict submax or submax+ to pair matching, since in practice such a priori restrictions would represent atypical use cases of both methods, where control units that could improve efficiency or covariate balance would be unnecessarily discarded. Instead, our study where we use HSR under pair matching and submax or submax+ under variable‐ratio matching highlight each procedure's respective strengths and provide a more honest, practice‐oriented assessment of them.

After matching, submax or submax+ splits the population L times into two subpopulations each, resulting in a total of K=2L+1 sharp null hypotheses. These include the sharp null hypothesis of no effect in the entire population and 2L hypotheses of no effect within in each subpopulation. For example, if sex (male or female) and APOE4 carriership (carrier or noncarrier) are specified as potential effect modifiers, then the method tests K=2×2+1=5 sharp null hypotheses of no effect among males, females, APOE4 carriers, APOE4 noncarriers, and the entire population.

Test statistics for each of the K=2L+1 null hypotheses are constructed by specifying a binary vector $c_{k}={\left(c_{1k},\ldots ,c_{Gk}\right)}^{⊤}$, where $c_{gk}\in \{0,1\}$, for k=1,…,K and g=1,…,G and the test statistic $T_{g}$ computed from each of the $G=2^{L}$ subgroups of matched sets. Specifically, for each Γ≥1 and k=1,…,K, the studentized test statistic is defined as

$D_{\Gamma k}=(\sum_{g=1}^{G}c_{gk}T_{g}-\theta_{\Gamma k})/\sigma_{\Gamma k}$, where $\theta_{\Gamma k}$ and $\sigma_{\Gamma k}$ denote the expectation and standard deviation under the sharp null hypothesis, ensuring that $\sum_{g=1}^{G}c_{gk}T_{g}$ is standardized to have mean zero and variance one; see Section 2.2 of Lee et al. [28] for details. In the example with gender and APOE4 carriership as binary effect modifiers, the population is divided into $G=2^{2}=4$ subgroups: male carriers (g=1), male noncarriers (g=2), female carriers (g=3), and female noncarriers (g=4), each with an associated test statistic $T_{g}$. The test statistic for the sharp null hypothesis of no effect among males (k=1) is based on the contrast vector $c_{1}=(1,1,0,0)$, corresponding to the sum of the statistics for male carriers and male noncarriers: $\sum_{g=1}^{4}c_{g1}T_{g}=T_{1}+T_{2}$.

Under the sharp null hypothesis of no effect for all matched sets, and for a given Γ≥1, the distribution of $D_{\Gamma }={\left(D_{\Gamma 1},\ldots ,D_{\Gamma K}\right)}^{⊤}$ converges to a normal distribution with mean 0 and covariance matrix $\rho_{\Gamma }$. We reject the sharp null of no effect in favor of a nonnegative treatment effect if $D_{\Gamma \max}=\max_{1\leq k\leq K}D_{\Gamma k}$ exceeds the critical value $\kappa_{\Gamma ,\alpha }$, the 1−α quantile of the distribution of $D_{\Gamma \max}$. We used the submax R package [36] to compute $D_{\Gamma }$ and the critical value $\kappa_{\Gamma ,\alpha }$. For two‐sided tests, the procedure can be adapted by performing two one‐sided tests and rejecting the null if either is rejected at level α/2.

Suppose that the sharp null hypothesis of no effect for all matched sets is rejected. For each k=1,…,K, define ${𝒮}_{k}$ as the set of sets in the kth comparison, that is, ${𝒮}_{k}{=⋃}_{g:c_{gk}=1}s_{g}$. For 𝒥⊆{1,…,K}, let $H_{𝒥}$ denote the sharp null hypothesis of no treatment effect in the combined subpopulations ${⋃}_{k\in 𝒥}{𝒮}_{k}$. We reject $H_{𝒥}$ if $\max_{k\in 𝒥}D_{\Gamma k}\geq \kappa_{\Gamma ,\alpha }$, where $\kappa_{\Gamma ,\alpha }$ is the critical value from the |𝒥|‐dimensional normal distribution. We note that all hypotheses of the form $H_{𝒥}$ can be tested using the closed testing procedure [46] described in Section 2.3.

## 4 Simulation Study

We conduct a simulation study to compare the different methods for sensitivity analysis with respect to (i) statistical power and (ii) identification of effect modifiers. Consider a setting with I matched pairs, p covariates, and G mutually exclusive and exhaustive subgroups, with treatment effects $\beta ={\left(\beta_{1},\ldots ,\beta_{G}\right)}^{⊤}$. Data are generated as follows.

$$\begin{array}{ll} & \text{Covariates:}\;x_{k}∼\text{Bernoulli}(1/2),\;k=1,\ldots ,p\\ & \text{Subgroup:}\;x_{1}\;\text{for}\;G=2,\;(x_{1},x_{2})\;\text{for}\;G=4\\ & \text{Potential outcome:}\;r_{Cij}∼N(0,1),\;r_{Tij}=r_{Cij}+\beta_{g},\;\forall i\in s_{g},\\ & \;j=1,2,\;g=1,\ldots ,G\\ & \text{Treatment assignment:}\;\mathrm{Pr}(Z_{i1}=1,Z_{i2}=0)\\ & \;=\mathrm{Pr}(Z_{i1}=0,Z_{i2}=1)=1/2,\;i=1,\ldots ,I\\ & \text{Observed outcome:}\;R_{ij}=Z_{ij}r_{Tij}+(1-Z_{ij})r_{Cij},\\ & \;i=1,\ldots ,I,\;j=1,2 \end{array}$$

We set G to be a power of 2 to reflect that the subgroups are constructed by binary confounders x. For example, when $G=2^{2}=4$, the subgroups correspond to the four combinations of $\left(x_{1},x_{2}\right)$: (0,0), (0,1), (1,0), and (1,1). Each subgroup has its own treatment effect $\beta_{g}$. For instance, β=(0.6,0,0,0.3) corresponds to treatment effects of 0.6, 0, 0, and 0.3 in the four subgroups, respectively. In this setup, $x_{1}$ and $x_{2}$ are two effect modifiers, resulting in one subgroup with a strong effect, two with no effects, and the one with a small effect.

We vary (i) the number of matched pairs I=200,400,600, (ii) the number of covariates p=4,8,12,20, and (iii) the effect size. Specifically, for G=2, we set β=(0.6,0,0,0.3) (small), β=(0.8,0,0,0.4) (moderate), β=(0.9,0,0,0.3) (moderately large), and β=(1.2,0,0,0.4) (large). The remaining covariates act as potential confounders but do not modify the treatment effect. Consequently, the proportion of non‐effect modifiers varies from 50% (p=4) to 90% (p=20), allowing us to investigate how proportion of non‐effect modifiers affects performance. We remark that the effect sizes (i.e., β) were not based on a specific prior study; rather, they were chosen to span a range of plausible scenarios with varying degrees of treatment effect heterogeneity. These values cover both settings with subtle effect modification and those with sufficiently strong signals to be detected by different methods. They also lead to clear performance differences across methods, particularly in scenarios with limited sample sizes or a large number of covariates.

Since submax requires a predefined set of potential binary effect modifiers, we evaluate its performance under different initial choices: (1) submax: using all covariates; (2) submax+ : using only covariates selected by CART; (3) submax (best): using only the true effect modifiers; (4) submax (worst): using only covariates that are not true effect modifiers.

**FIGURE 3:**
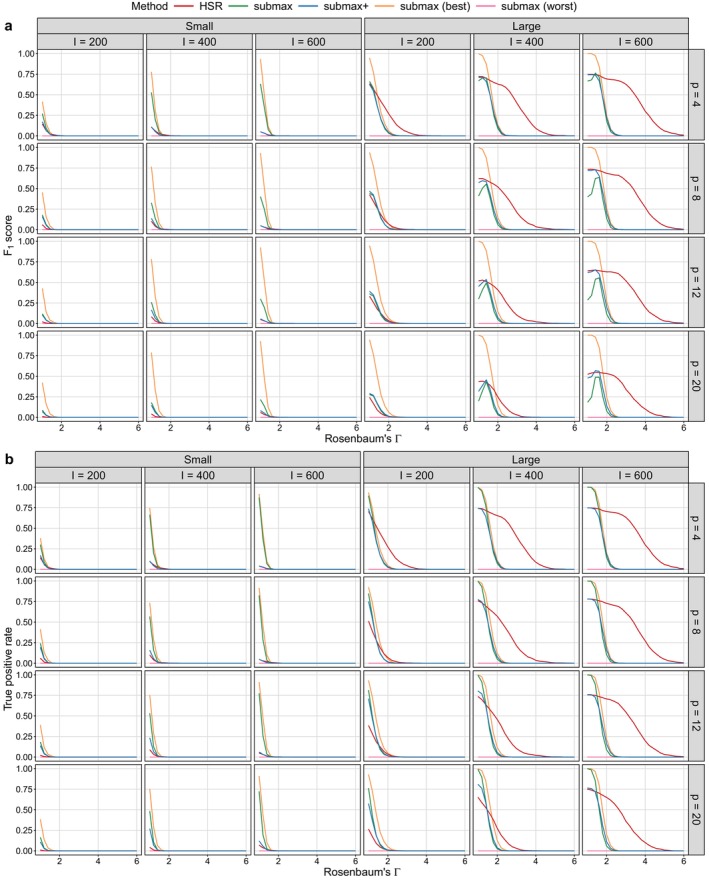
$F_{1}$ score and true positive rate (TPR) for hypothesis testing under different simulation settings with two binary effect modifiers. Columns correspond to effect size (small: β=(0.6,0,0,0.3); large: β=(1.2,0,0,0.4)) and the number of matched pairs (I), while rows correspond to the number of confounders (p). Sensitivity parameter values (Γ) range from 1 to 6 in increments of 0.2.

**FIGURE 4:**
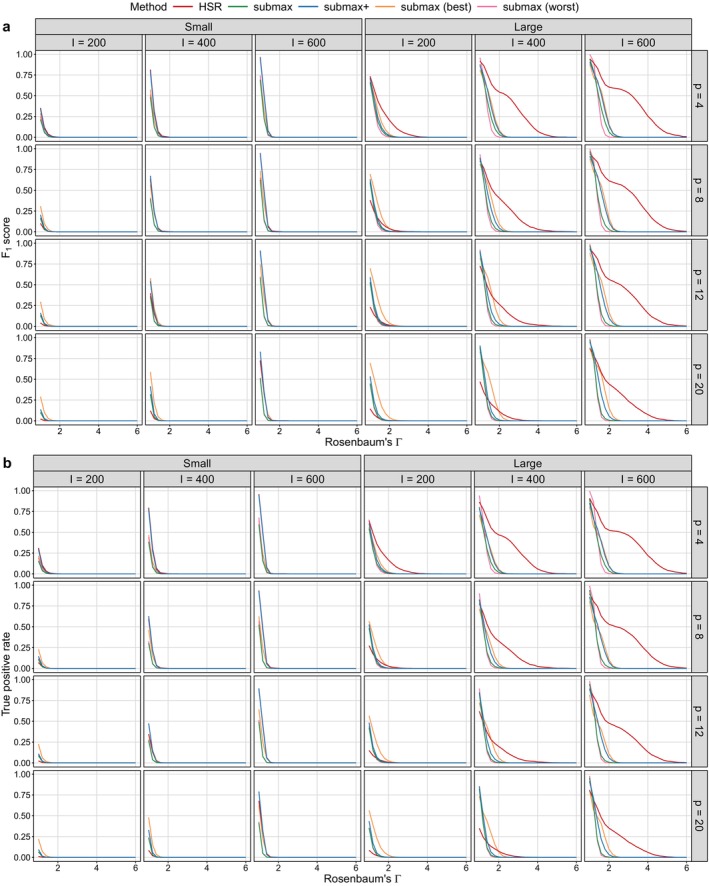
$F_{1}$ score and true positive rate (TPR) for effect modifier identification under different simulation settings with two binary effect modifiers. Columns correspond to effect size (small: β=(0.6,0,0,0.3); large: β=(1.2,0,0,0.4)) and the number of matched pairs (I), while rows correspond to the number of confounders (p). Sensitivity parameter values (Γ) range from 1 to 6 in increments of 0.2.

We conduct 1000 replicates for each simulation setting. For simplicity, we present the results for small and large effect sizes in Figures 3 and 4, where the performance differences between methods are more pronounced. Results for moderate and moderately large effect sizes, which exhibit more gradual performance changes, are provided in Appendix Section D. Additional results for a simpler setting with one binary effect modifier (G=2 subgroups) are reported in Appendix Section D and show patterns consistent with the main findings.

### 4.1 Statistical Power

For each method and each sensitivity parameter value Γ≥1, we assess the statistical power by the $F_{1}$ score and the true positive rate (TPR), defined as

$$\begin{array}{l} F_{1}\;\text{score}=\frac{2TP}{2TP+FP+FN},\;\text{TPR}=\frac{TP}{TP+FN}, \end{array}$$

where TP, FP, and FN denote the numbers of true positives, false positives, and false negatives, respectively. A true positive corresponds to the rejection of a false null hypothesis at significance level, and a false positive corresponds to the rejection of a true null hypothesis. Hypothesis rejection is determined using the closed testing procedure at level α=0.05.

The number of hypotheses tested varies across methods. For submax (best), five predefined contrasts are tested consistently across all simulation settings. In contrast, the number of hypotheses in submax and submax (worst) depends on the number of covariates p, amounting to 2p+1 and 2(p−2)+1 hypotheses, respectively. For both HSR and submax+ , the number of hypotheses is determined adaptively by the structure of the CART tree and can vary across replicates. If no split is selected, only one hypothesis is tested. If L≤p covariates are selected for splitting in CART, then 2L+1 hypotheses are evaluated.

Figure 3 provides a comprehensive view of how statistical power varies with three key design factors, while simultaneously illustrating the impact of the initial selection of potential effect modifiers within the submax framework. The two oracle methods, submax (best) and submax (worst), provide clear and interpretable benchmarks to compare the other methods. Also, across all methods, power increases with effect size and sample size, but declines as the number of covariates increases.

The performance of HSR is highly sensitive to both the sample size and the number of covariates, as reflected by the pronounced shift of the red lines across the columns and rows of Figure 3. HSR performs the worst when sample size is small (e.g., I=200) and the number of covariates is moderate to large (e.g., p≥8). As the sample size increases and the number of covariates decreases, its performance gradually improves. When the sample size is sufficiently large (e.g., I≥400) and the number of covariates is small to moderate (e.g., p≤12), HSR achieves the best performance under scenarios with large effect sizes (right panel in Figure 3) and performs comparably to submax+ under small effect sizes (left panel in Figure 3).

When the effect size is small (left panel in Figure 3), submax+ consistently outperforms both submax and HSR, regardless of sample size (columns of Figure 3) or number of covariates (rows of Figure 3). The power of submax is particularly low in this setting, as shown by the green lines overlapping with the pink lines representing the worst case, submax (worst). This is likely due to its reduced ability to reject false null hypotheses when many false null hypotheses are tested and the effect size is small. Although submax+ and HSR test the same number of hypotheses, submax+ achieves a consistently higher $F_{1}$ score, indicating better overall power when the signal is weak. When the effect size is large (right panel in Figure 3), submax+ continues to outperform submax, with the gap between the green and blue lines becoming more pronounced as the sample size increases. Notably, HSR can exceed the performance of submax+ when the sample size is sufficiently large (e.g., I≥400) and the number of covariates is small to moderate (e.g., p≤12).

Taken together, these results suggest a hierarchy of practical recommendations. submax+ and HSR are generally preferable for maximizing statistical power. In studies with limited sample size or small effect size, submax+ provides the most reliable performance. When the sample size is sufficiently large (e.g., I≥400) or the proportion of non‐effect modifiers is relatively low (e.g., ≤25%), HSR can outperform submax+ . We also note that our findings are broadly consistent with Lee et al.'s [28] recommendation to use submax when effect sizes are moderate. Indeed, increasing the effect size leads to improved performance of submax, as demonstrated by the comparison between the left and right panels in Figure 3. However, it still underperforms relative to submax+ and HSR. This gap arises because both submax+ and HSR incorporate a data‐driven selection of potential effect modifiers prior to testing, which is critical in regimes where effect sizes are small or the number of potential effect modifiers greatly exceeds the number of true modifiers.

### 4.2 Detecting True Effect Modifiers

For each method and each sensitivity parameter value Γ≥1, we assess the identification of effect modifiers by the $F_{1}$ score and the TPR, defined as

$$\begin{array}{l} F_{1}\;\text{score}=\frac{2TP}{2TP+FP+FN},\;\text{TPR}=\frac{TP}{TP+FN}, \end{array}$$

where TP, FP, and FN denote the numbers of true positives, false positives, and false negatives, respectively. A true positive corresponds to identifying a true effect modifier, and a false positive corresponds to identifying a non‐effect modifier.

For HSR, a covariate is considered an effect modifier if the CART tree splits on that variable and one of the resulting child node null hypotheses is rejected using the closed testing procedure at level α=0.05. If no split is selected, no covariates are tested, and consequently, no effect modifiers are identified. For submax, submax+ , submax (best), and submax (worst), a covariate is considered an effect modifier if the method rejects a null hypothesis corresponding to a subpopulation defined by that variable using the closed testing procedure at level α=0.05.

Figure 4 provides a comprehensive view of how effect modifier identification varies with three key design factors, while simultaneously illustrating the impact of the initial selection of potential effect modifiers within the submax framework. submax (best) always starts with the two true effect modifiers, whereas submax (worst) yields zero $F_{1}$ score and TPR because none of the true effect modifiers are included in the tested set. Across all methods, $F_{1}$ score increases with effect size and sample size, but declines as the number of covariates increases.

submax consistently achieves a higher TPR (Figure 4b) than submax+ , approaching the best‐case scenario, submax (best), when either the effect size or the sample size is large. This is demonstrated by the narrow gap between the green and orange lines in Figure 4b. However, $F_{1}$ score of submax (green lines in Figure 4a) remains substantially low, and lower than that of submax+ when the effect size is large. This pattern suggests that leveraging variables selected by the CART tree improves the identification of true effect modifiers in submax, particularly by reducing false positives when the number of covariates is moderate to large (e.g., p≥8).

When the effect size is small (left panel in Figure 4), both submax and submax+ outperform HSR, with the performance gap becoming more evident at smaller sample sizes (e.g., I=200). Although submax achieves a higher $F_{1}$ score than both HSR and submax+ , this advantage diminishes as the sample size decreases (columns of Figure 4) and the number of covariates increases (rows of Figure 4), resulting in comparable performance between submax and submax+ .

When the effect size is large (right panel in Figure 4), HSR exhibits a slower decay of $F_{1}$ score compared to submax and submax+ . HSR particularly benefits from larger sample sizes. When I=200, it performs comparably to or worse than both submax and submax+ . However, as the sample size increases, HSR achieves the best performance once I reaches 400, as indicated by the pronounced upward shift of the red lines across rows in Figure 4.

Overall, these results suggest that if the goal is to identify true effect modifiers even at the expense of some false positives, then submax is a suitable choice. If the goal is to have low false positives, at the expense of some decrease in TPR, HSR generally outperforms submax when the effect size or the sample size is large. In settings where the effect size is small and the sample size is limited, submax+ offers low false positives at the expense of some decrease in TPR.

## 5 Results from Data Analysis

### 5.1 Sensitivity Analysis With HSR

Based on prior works, we considered five potential effect modifiers: sex (male or female) [8, 13], copies of APOE ε4 allele (0, 1, or 2) [14, 15], family history of dementia or and AD (no history, history of dementia, or history of AD) [16, 17, 18, 19], years of education (high school, college, graduate) [51, 52], and the tertiles of the age groups at baseline (55 to 71, 72 to 77, or 78 to 91 years old) [53, 54]. I=295 matched pairs were formed with almost exact matching on these five covariates and near‐fine balance on copies of APOE2, marital status, and quantiles of PRS.

Before matching, a couple of covariates had absolute standardized differences above 0.1, notably sex, residence type, and PRS for chronotype. After matching, all 31 covariates had absolute standardized differences that were less than 0.1.

**FIGURE 5:**
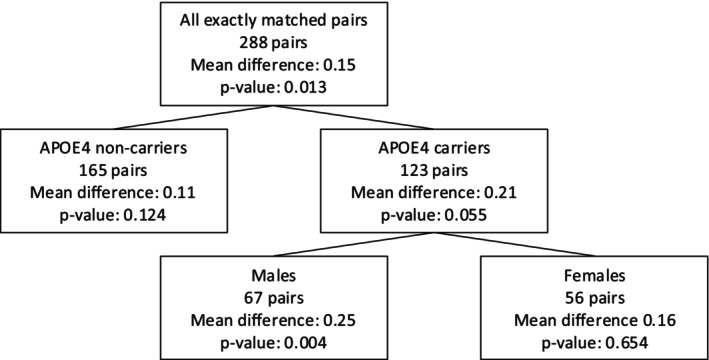
The estimated regression tree with 288 matched pairs. Initially, the tree was estimated using 277 matched pairs that were exactly matched for five potential effect modifiers (sex, APOE4, family history of dementia and AD, years of education, and age at baseline). We then rematched matched pairs to be exactly matched for APOE4 and sex, leading to a total of 288 matched pairs. The average treatment effect within each subgroup (i.e., mean difference) and the p‐value when Γ=1.

The regression tree in Figure 5 was constructed using 277 matched pairs that are exactly matched for all five possible effect modifiers. The regression tree split on two covariates, APOE4 carriership and sex, defining G=3 subgroups: APOE4 noncarriers (Group 1), male APOE4 carriers (Group 2), and female APOE4 carriers (Group 3). Then 18=295−277 inexactly matched pairs were re‐paired to be exactly matched for APOE4 carriership and sex. Ultimately, 288 matched pairs were used for the subsequent sensitivity analysis.

At Γ=1 where there is no unmeasured confounding, the two‐sided test rejected the sharp null hypothesis of no treatment effect for all 288 matched pairs at level α=0.05. We used the weighted M‐test with weights appropriate for long‐tailed distributions, specifically inner and outer trimming of 0 and 3, λ=1/2, and $\left(m,\underline{m},\bar{m})=(20,12,19\right)$. Table 2 shows the results for testing the hypothesis of no treatment effect in the subgroups defined by the estimated tree and using closed testing to account for multiple testing. At Γ=1.17, the closed testing rejects the hypothesis of no treatment effect for male APOE4 carriers. This implies that the effect of sleep problems on the decline of EF among male APOE4 is nullified by an unmeasured confounder that changes the odds of having sleep problems by Γ>1.17. Also, according to the amplification analysis, a bias of Γ=1.17 can correspond to an unobserved covariate that doubles the odds of having sleep problems and increases the odds of changing the decline in EF score by a factor of 1.6.

**TABLE 2:** Sensitivity analysis using the CART method. Individual groups refer to the three groups in Figure 5 where Group 1 is the subgroup of APOE4 noncarriers, Group 2 is the subgroup of male APOE4 carriers, and Group 3 is the subgroup of female APOE4 carriers. Testing for the overall and the two groups uses the truncated product of p‐values. Each entry represents the p‐value for testing the sharp null hypothesis of no treatment effect among groups defined by the column. Bold represents the smallest Γ that exceeds the familywise error rate of 0.05 via the closed testing procedure.

Γ	Overall	Two groups			Individual groups		
	G1×G2×G3	G1×G2	G1×G3	G2×G3	G1	G2	G3
1.00	0.019	0.011	1.000	0.011	0.124	0.004	0.654
1.10	0.032	0.020	1.000	0.020	0.261	0.009	0.837
1.17	**0.047**	**0.031**	1.000	**0.031**	0.392	**0.015**	0.960
1.18	0.050	0.033	1.000	0.033	0.412	0.016	0.977
1.20	0.056	0.036	1.000	0.036	0.455	0.018	1.000
1.30	0.091	0.061	1.000	0.061	0.687	0.031	1.000
1.40	0.140	0.096	1.000	0.096	0.932	0.049	1.000

### 5.2 Sensitivity Analysis With Submax+

Since the CART tree split on sex and APOE carriership, sex and APOE carriership were treated as potential effect modifiers for submax+. This yielded four subgroups: males, females, APOE4 carriers, and APOE4 noncarriers. These subgroups were then tested in submax+ . We remark that CART dichotomized APOE ε4 status into carriers (one or two copies) and noncarriers (zero copies). Also, sex was measured as a binary variable, either male or female. We created I=295 variable‐ratio matched sets, comprising 295 treated individuals and 661 controls. Almost exact matching was performed on sex and APOE4 carriership. Additionally, near‐fine balance constraints were imposed on paternal history of dementia, marital status, and residence type. After variable‐ratio matching, all 31 covariates had absolute standardized differences below 0.05.

Table 3 presents results for testing the sharp null hypothesis of no treatment effect at various values of Γ≥1. We use the equally weighted M‐test with inner and outer trimming parameters of 1.5 and 2.6, respectively, and λ=1/2. At Γ=1, corresponding to no unmeasured confounding, the sharp null hypothesis of no effect for all matched sets is rejected at level α=0.05. This rejection holds until Γ>1.81, indicating that the causal effect of sleep problems on EF decline would be invalidated by an unmeasured confounder that increases the odds of treatment assignment by more than 1.81. According to the amplification analysis, such a bias corresponds to an unobserved covariate that triples the odds of having sleep problems and increases the odds of changing the decline in EF score by a factor of 3.7.

**TABLE 3:** Sensitivity analysis using the submax method. Each entry refers to the value of test statistics at different values of Γ≥1, and k refers to the comparison groups. Deviates that are larger than the critical value $\kappa_{\Gamma ,\alpha }$ for α=0.05 are in bold. The average treatment effect within each subgroup (i.e., mean difference) was computed when Γ=1.

k	1	2	3	4	5	
Subpopulation	All	Female	Male	APOE4 carriers	APOE4 noncarriers	Maximum
	$D_{\Gamma 1}$	$D_{\Gamma 2}$	$D_{\Gamma 3}$	$D_{\Gamma 4}$	$D_{\Gamma 5}$	$D_{\Gamma max}$ (p‐values)
# Matched sets	295	134	152	126	160	
(Sample size)	(956)	(393)	(520)	(418)	(498)	
Mean difference	0.17	0.15	0.20	0.21	0.13	
Γ=1.00	**4.25**	2.36	**3.79**	**3.43**	**2.71**	**4.25** (0.000)
Γ=1.20	**3.71**	2.02	**3.36**	**3.02**	2.36	**3.71** (0.000)
Γ=1.40	**3.25**	1.73	**3.00**	**2.68**	2.07	**3.25** (0.003)
Γ=1.60	**2.86**	1.48	**2.70**	**2.38**	1.83	**2.86** (0.008)
Γ=1.80	**2.51**	1.26	**2.43**	2.12	1.62	**2.51** (0.023)
Γ=1.81	**2.49**	1.25	**2.42**	2.11	1.61	**2.49** (0.025)
Γ=1.82	2.48	1.24	2.41	2.10	1.60	2.48 (0.026)

The closed testing procedure rejects the sharp null hypothesis of no effect in the subpopulation of males at Γ=1, suggesting a causal effect of sleep problems on EF decline among males. This causal conclusion remains valid even in the presence of an unmeasured confounder that increases the odds of treatment assignment by up to Γ=1.75, after adjusting for multiple testing. According to the amplification analysis, such a bias corresponds to an unobserved covariate that triples the odds of having sleep problems and increases the odds of change in EF score by a factor of 3.4.

### 5.3 Multiple Linear Regression

Finally, for comparison with the methods above, we consider multiple linear regression models regressing the outcomes $R_{ij}$ on the treatment indicator $Z_{ij}$ and all measured covariates $x_{ij}$:

(1) $$\begin{array}{ll} R_{ij} & =\alpha +\beta Z_{ij}+\gamma_{1}x_{ij1}+\cdots +\gamma_{p}x_{ijp}+\varepsilon \end{array}$$

(2) $$\begin{array}{ll} R_{ij} & =\alpha +\beta Z_{ij}+\gamma_{1}x_{ij1}+\cdots +\gamma_{p}x_{ijp}+\eta_{1}Z_{ij}x_{ij1}\\ & \;+\cdots +\eta_{p}Z_{ij}x_{ijp}+\varepsilon \end{array}$$

(3) $$\begin{array}{ll} R_{ij} & =\alpha +\beta Z_{ij}+\gamma_{1}x_{ij1}+\cdots +\gamma_{p}x_{ijp}+\eta_{1}Z_{ij}x_{ijm_{1}}\\ & \;+\cdots +\eta_{L}Z_{ij}x_{ijm_{L}}+\varepsilon \end{array}$$

Model (1) specifies a regression model without effect modification. Model (2) considers effect modification through interaction terms between the treatment indicator $Z_{ij}$ and all measured covariates $x_{ij}$. Model (3) includes effect modification only for the L variables selected by the CART tree, where $m_{1},\ldots ,m_{L}\in \{1,\ldots ,p\}$ indexes these selected variables. We remark that none of these regression models account for unmeasured confounding.

Under model (1), the estimated coefficient for sleep problems is 0.10 (p=0.031, 95% CI: (0.01,0.18)), indicating that individuals with sleep problems experience a greater decline in EF scores compared to those without sleep problems. In models (2) and (3), which explore effect heterogeneity, the estimated coefficients for the interaction terms involving sleep problems were not significant at the α=0.05 level after applying a Bonferroni correction for multiple testing. Even without Bonferroni correction, the smallest p‐value from both interaction models was 0.010.

## 6 Discussion

**FIGURE 6:**
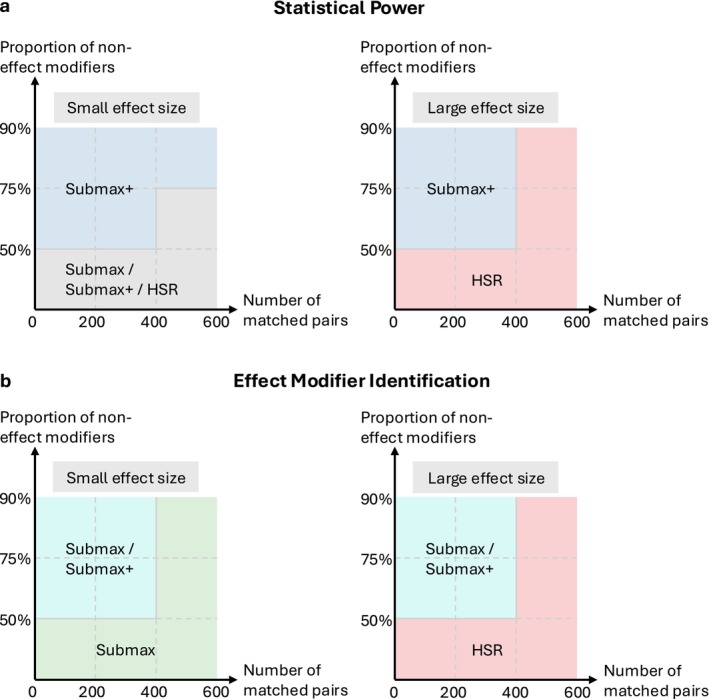
Recommendation of methods based on effect size, sample size, and the proportion of non‐effect modifiers. The proportion of non‐effect modifiers is calculated as one minus the number of true effect modifiers divided by the number of total covariates. For definitions of small and large effect sizes, see Figures 4 and 5. The figure summarizes simulation results on statistical power and effect modifier identification.

We compared different methods for sensitivity analysis of heterogeneous treatment effects in the presence of unmeasured confounding in order to provide concrete guidance to practitioners on selecting robust procedures tailored to their study designs. While no simulation study can capture every practical scenario, our results highlight how method performance depends on three key factors: (i) sample size, (ii) the number of covariates and the proportion of true effect modifiers among them, and (iii) the expected effect size. These factors jointly determine how submax, submax+ , and HSR perform across realistic settings, and the overall recommendation is summarized in Figure 6.

When the investigator has limited prior knowledge and faces a large set of covariates, the choice between submax and submax+ depends on the analytic goal. If the goal is to maximize effect modifier identification, submax tends to achieve the highest TPR, but at the expense of more false positives and lower $F_{1}$. If the goal is to maximize statistical power while controlling Type I error rates, submax+ provides more stable performance, particularly when the effect size or the sample size is small. When the investigator has some prior knowledge about the proportion and effect size of true modifiers, the choice becomes more straightforward. If the sample size is sufficiently large or the proportion of non‐effect modifiers is low, we recommend HSR for studies with expected large effect sizes and submax for expected small to moderate effect sizes. In regimes where the sample size is small and the proportion of non‐effect modifiers is high, we generally recommend submax+.

The real data analysis complements our recommendations mentioned above. Specifically, HSR and submax+ both detected a causal effect of sleep problems on decline in EF, with effect estimates varying by APOE4 carriership and sex. Submax+ revealed heterogeneous treatment effects in males and APOE4 carriers separately, while HSR highlighted the subgroup of male APOE4 carriers. The evidence from submax+ remains valid up to Γ=1.81, which is greater than Γ=1.17 obtained from HSR.

This difference may reflect the better covariate balance and larger effective sample size achieved by submax+. In particular, variable‐ratio matching matches one treated individual to multiple controls and achieves better covariate balance than pair matching. With variable‐ratio matching, all absolute standardized differences remain below 0.05, whereas pair matching yields differences up to 0.10. HSR is confined to pair matching, while submax+ accommodates both pair and variable‐ratio schemes, thereby increasing the number of matched samples. Moreover, submax+ allows some sets to be inexactly matched, whereas HSR requires exact matches as defined by the terminal leaves of the estimated tree.

We briefly remark that the methods discussed in the paper are nonparametric, adjusting for observed covariates through optimal matching, with submax+ and HSR further relying on CART for subgroup identification. Although we focus on continuous outcomes for simplicity, none of the methods are restricted to this setting. In addition, our conclusions are based on a workflow where the potential subgroups are first discovered under the assumption of no unmeasured confounding (Γ=1) and the treatment effects within these subgroups are tested under different magnitudes of unmeasured confounding (Γ>1). It would be interesting to study the sensitivity of heterogeneous treatment effects when the potential subgroups are discovered under different assumptions about unmeasured confounding and the sensitivity of the treatment effects for these discovered subgroups.

We highlight several key limitations of our analysis. First, the formation of matched sets depended on various parameters, such as the caliper for matching, near‐fine balance constraints, the selection of covariates for exact matching, and the depth of the CART tree. Notably, the estimated tree structure may vary substantially with different tuning parameters. However, as shown by Hsu et al. [27], as long as the tree is constructed using the absolute differences in outcomes within each pair, the subsequent inference on the sharp null hypothesis of no effect remains valid. Second, our sample is not representative of all adults at risk for dementia and AD, particularly individuals from non‐white racial groups. Prior studies have demonstrated that race can influence cognitive decline among individuals with MCI and AD [55]. Given that the ADNI cohort is predominantly white, our findings may not generalize well to non‐white populations. Third, although definitions of sleep problems vary (see Appendix Section B), our definition of abnormal sleep patterns differs from those used in previous studies. Nevertheless, we obtained similar conclusions, supporting the established association between sleep problems, cognitive impairment, and increased risk of dementia.

## Funding

The authors have nothing to report.

## Conflicts of Interest

The authors declare no conflicts of interest.

## Supporting information


**Data S1**: Additional supporting information may be found in the online version of the article at the publisher's website.

## Figures and Tables

**FIGURE D1:**
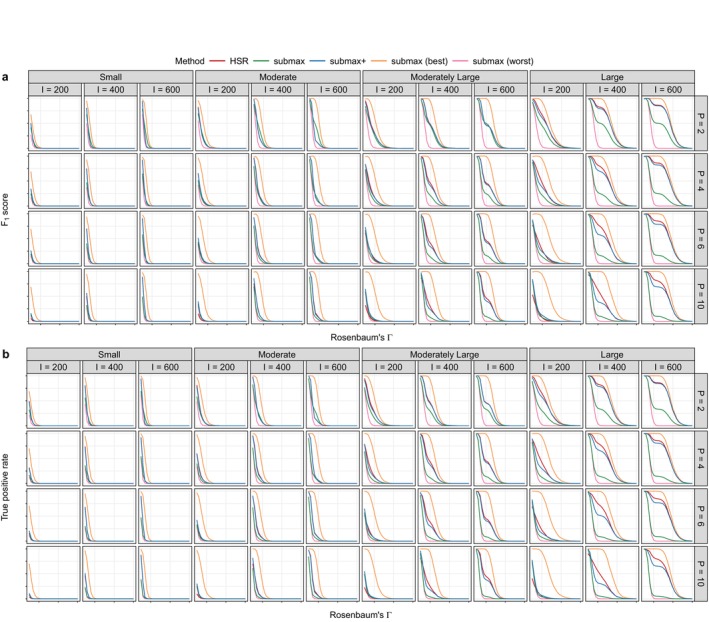
$F_{1}$ score and true positive rate (TPR) for hypothesis testing under different simulation settings with one binary effect modifier. Columns correspond to treatment effect sizes (small: β=(0.5,0), moderate: β=(0.7,0), moderately large: β=(0.9,0), and large: β=(1.0,0)) and the number of matched pairs (I). Rows correspond to the number of confounders (p). Sensitivity parameter values (Γ) range from 1 to 6 in increments of 0.2.

**FIGURE D2:**
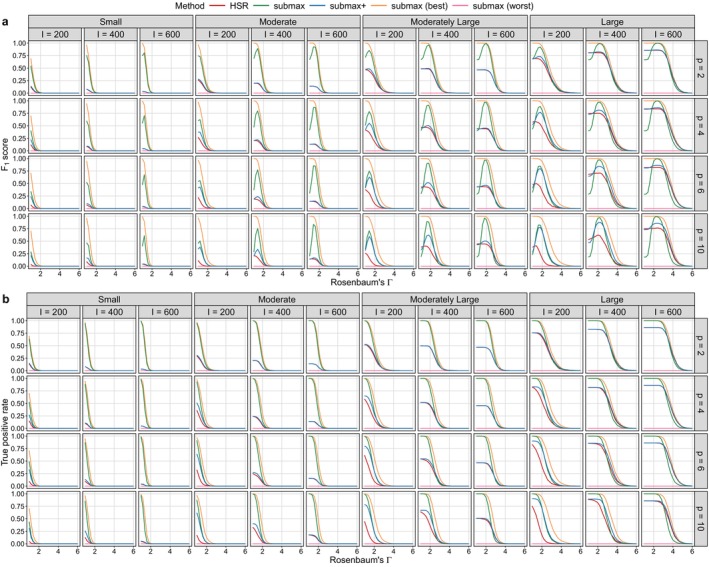
$F_{1}$ score and true positive rate (TPR) for effect modifier identification under different simulation settings with one binary effect modifier. Columns correspond to treatment effect sizes (small: β=(0.5,0), moderate: β=(0.7,0), moderately large: β=(0.9,0), and large: β=(1.0,0)) and the number of matched pairs (I). Rows correspond to the number of confounders (p). Sensitivity parameter values (Γ) range from 1 to 6 in increments of 0.2.

**FIGURE D3:**
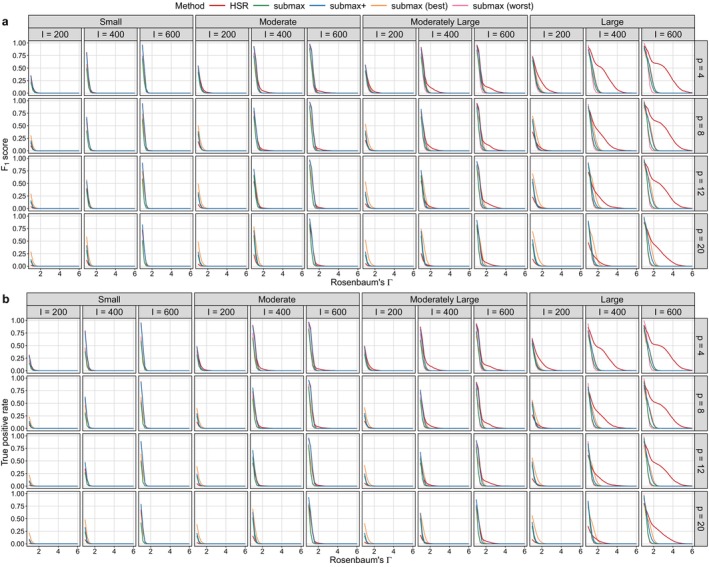
$F_{1}$ score and true positive rate (TPR) for hypothesis testing under different simulation settings with two binary effect modifiers. Columns correspond to treatment effect sizes (small: β=(0.6,0,0,0.3), moderate: β=(0.8,0,0,0.4), moderately large: β=(0.9,0,0,0.3), and large: β=(1.2,0,0,0.4)) and the number of matched pairs (I). Rows correspond to the number of confounders (p). Sensitivity parameter values (Γ) range from 1 to 6 in increments of 0.2.

**FIGURE D4:**
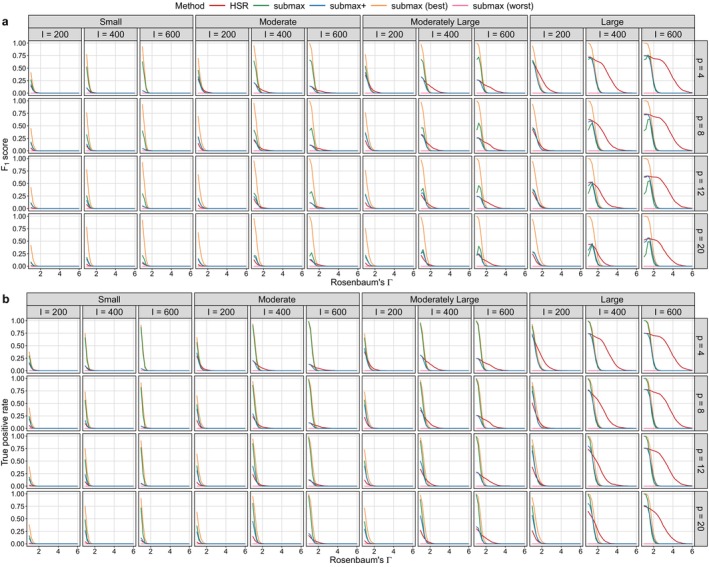
$F_{1}$ score and true positive rate (TPR) for effect modifier identification under different simulation settings with two binary effect modifiers. Columns correspond to treatment effect sizes (small: β=(0.6,0,0,0.3), moderate: β=(0.8,0,0,0.4), moderately large: β=(0.9,0,0,0.3), and large: β=(1.2,0,0,0.4)) and the number of matched pairs (I). Rows correspond to the number of confounders (p). Sensitivity parameter values (Γ) range from 1 to 6 in increments of 0.2.

**FIGURE D5:**
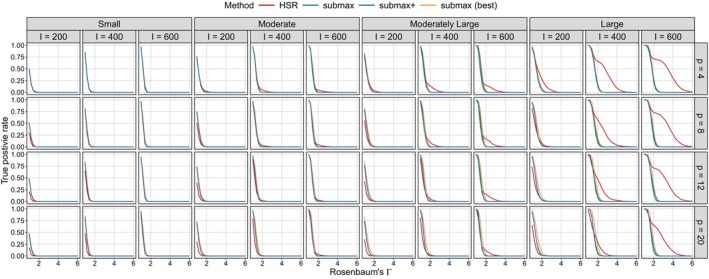
True positive rate (TPR) for testing global null hypothesis under different simulation settings. Columns correspond to treatment effect sizes (small: β=(0.6,0,0,0.3), moderate: β=(0.8,0,0,0.4), moderately large: β=(0.9,0,0,0.3), and large: β=(1.2,0,0,0.4)) and the number of matched pairs (I). Rows correspond to the number of confounders (p). Sensitivity parameter values (Γ) range from 1 to 6 in increments of 0.2.

## Data Availability

The data that support the findings of this study are available from the Alzheimer's Disease Neuroimaging Initiative (ADNI). Restrictions apply to the availability of these data, which were used under license for this study. Data are available from https://adni.loni.usc.edu/ with the permission of Alzheimer's Disease Neuroimaging Initiative (ADNI).

## Appendix A Trimmed, Weighted M‐Statistics

The weighted M‐statistic with inner trimming, an extension of Maritz's version [41] of Huber's [42] M‐statistic, is proposed by Rosenbaum [43, 44, 45] to increase the power of a sensitivity analysis. Under Fisher's sharp null of no effect, $R_{ij}=r_{Cij}$, $R_{ij}-R_{il}=r_{Cij}-r_{Cil}$, and the weighted M‐statistic with inner trimming becomes

(A1)
$$\begin{array}{cc} T_{g} & =\underset{i\in s_{g}}{\sum }w_{i}\overset{n_{i}}{\underset{j=1}{\sum }}Z_{ij}\overset{n_{i}}{\underset{l=1}{\sum }}\psi_{\text{in}}\{(r_{Cij}-r_{Cil})/s\}\\ & =\underset{i\in s_{g}}{\sum }w_{i}\overset{n_{i}}{\underset{j=1}{\sum }}Z_{ij}q_{ij},\;q_{ij}=\overset{n_{i}}{\underset{l=1}{\sum }}\psi_{\text{in}}\{(r_{Cij}-r_{Cil})/s\}, \end{array}$$

where

- $w_{i}$ is nonnegative with wi∝w˜i=∑l=m_m‾ai−1l−1Ig−aim−l, where $\left(m,\underline{m},\bar{m}\right)$ are weights with $1\leq \underline{m}\leq \bar{m}\leq m$ and $a_{i}$ is the rank of the ranges $\max_{1\leq j\leq n_{i}}q_{ij}-\min_{1\leq l\leq n_{i}}q_{il}$ with $q_{ij}$ defined in (A1); refer to Section 4.2 in Rosenbaum [44] for details.
- $\psi_{\text{in}}(y)=\{h/(h-\iota )\}\cdot \text{sign}(y)\cdot \max\{0,\;\min(|y|,\;h)-\iota \}$, where the terms h and ι are outer and inner trimming with 0≤ι<h and $\psi_{\text{in}}(0)=0$; see Section 6.2 in Rosenbaum [45] for details.
- s is the λ quantile of all pairwise absolute differences $\left|R_{ij}-R_{il}\right|$, $1\leq j<l\leq n_{i}$, $i\in s_{g}$ with 0<λ<1. For example, λ=1/2 yields the median of the absolute difference and s is the value of the median.

## Appendix B Literature Review of Sleep Problems

Sleep problems have been defined in various ways in the literature, including, but not limited to, reduced slow‐wave sleep and rapid eye movement sleep [11], sleep fragmentation [9], sleep‐disordered breathing [6], and both short [2] and long sleep duration [4]. The definitions of sleep problems often depend on the specific phenomena that the investigator aims to study.

Sleep quality is often considered a symptom or marker of sleep problems. Although it is an important and modifiable factor in sleep research, its definition also varies across studies [56, 57, 58]. For example, a recent meta‐analysis of 65 randomized controlled trials [59] examined the causal effects of sleep‐improving interventions on mental health outcomes. In this study, sleep quality was assessed based on sleep continuity and daytime functioning, but as the authors note, “…different sleep disorders have different conceptualisations of improvement that might not include sleep quality.”

In our study, the definition of sleep problems was based on data availability and a recent recommendation [58], which suggested using self‐reported assessments from different perspectives, such as self‐reported and caregiver‐reported sleep issues. Section 6 further discusses how to interpret our results within the broader context of other research on sleep problems and cognitive decline.

## Appendix C Further Details of Data Analysis

### Processing of Genetic Data

To adjust for potential genetic confounding, we constructed PRS for insomnia, chronotype, and sleep duration. Specifically, we followed the procedure outlined in Zhao et al. [60] and used PRSice‐2 [61] to compute the PRS. The genome‐wide association study (GWAS) summary statistics used to generate the PRS were first pruned using PLINK [62]. We set the linkage disequilibrium (LD) block window to 100 variants, with a step size of 5 variants to shift the window, and applied a pairwise LD ($r^{2}$) threshold of 0.1. LD estimation was based on European‐ancestry samples from the 1000 Genomes Project Phase III, which served as the reference panel.

### Study Population

Table C1 summarizes covariates, exposure, and outcome between the analysis sample (n=1131) and the original sample (n=2430). Overall, most covariates between the analysis sample and the original sample are similar, except for five covariates. Specifically, the proportion of females is slightly lower in the analysis sample (43.8%) than in the original sample (46.8%). The proportion of individuals identifying as Hispanic or Latino is slightly lower in the analysis sample (1.3%) than in the original sample (3.6%). The analysis sample has fewer missing values for family history than the original sample. The analysis sample shows higher baseline composite memory score (0.36) and EF score (0.30) than those in the original sample (0.23 and 0.15, respectively). But the outcome, which is defined as the change from baseline measurement, is similar in both samples. We remark that the analysis sample does not have missing values for genetic data (PRS and APOE gene), which is expected since our analysis sample only considers individuals who have their genetic data measured.

**TABLE C1:** Summary statistics of 31 covariates in the analysis sample and the original sample.

Covariates	Analysis sample		Original sample	
	(*n* = 1131)		(*n* = 2430)	
	Mean (SD)	%Missing	Mean (SD)	%Missing
Sex (Female)	43.8%		46.6%	0.5%
Latino ethnicity	1.3%		3.6%	0.5%
Age at baseline, years	73.68 (7.04)		73.00 (7.54)	0.5%
Retirement age, years	74.10 (23.91)	1.5%	74.65 (24.58)	2.5%
Education, years	16.07 (2.76)		15.98 (2.82)	0.5%
Right‐handed	91.4%		91.2%	0.5%
Residence type				
House	77.3%		75.2%	0.5%
Condo/co‐op	12.3%		12.1%	0.5%
Rented apartment	5.0%		7.7%	0.5%
Retirement community	3.0%		2.7%	0.5%
Mobile home	1.1%		0.9%	0.5%
Assisted living	0.4%		0.5%	0.5%
Marital status				
Married	77.6%		74.4%	0.5%
Widowed	10.9%		11.1%	0.5%
Divorced	8.0%		9.7%	0.5%
Never married	3.1%		4.2%	0.5%
Family history				
Mom with dementia	40.6%	2.3%	40.6%	8.6%
Mom with AD	26.9%	8.7%	25.9%	16.3%
Dad with dementia	18.4%	4.6%	18.3%	10.8%
Dad with AD	10.1%	8.0%	9.7%	14.4%
Number of female siblings	1.17 (1.27)	0.6%	1.22 (1.32)	6.4%
Number of siblings w/o dementia and AD	2.05 (1.99)	0.6%	2.15 (2.04)	6.4%
Number of siblings with dementia	0.06 (0.28)	0.6%	0.05 (0.26)	6.4%
Number of siblings with AD	0.15 (0.46)	0.6%	0.13 (0.45)	6.4%
APOE ε2 copies (0/1/2)	90.5/9.5/0.0%		91.1/8.8/0.1%	37.9%
APOE ε4 copies (0/1/2)	54.4/36.1/9.5%		52.6/37.5/9.9%	37.9%
Polygenic risk scores ($\times 10^{-6}$)				
Insomnia	−6.08 (20.01)		−5.99 (19.63)	37.9%
Sleep duration	84.58 (8.45)		84.52 (8.35)	37.9%
Chronotype	−39.87 (8.41)		−39.85 (8.45)	37.9%
Baseline composite memory score	0.34 (0.86)		0.23 (0.90)	32.1%
Baseline composite executive function score	0.31 (1.00)		0.15 (1.04)	32.1%
Exposure: Sleep problems	26.1%		29.8%	25.6%
Outcome: Decline in composite executive function score	0.10 (0.69)		0.09 (0.69)	47.2%

The prevalence of the exposure, sleep problems, is slightly lower in the analysis sample (26.1%) compared to the original sample (29.8%). Note that the exposure and outcome variables have a large proportion of missing data in the original sample, which is expected since our analysis sample only considered individuals who had their exposure and outcome measured.

## Appendix D Further Details of Simulation Study

### Simulation Results With a Single Effect Modifier

In the main text, we present results for scenarios with two binary effect modifiers. In addition, we conducted a simulation study with a single binary effect modifier under similar settings. Specifically, G=2 subgroups are defined by $x_{1}=0$ and $x_{1}=1$. The results for hypothesis testing and effect modifier identification are shown in Figures D1 and D2, respectively. Specifically, we vary (i) the number of matched pairs, I∈200,400,600, (ii) the number of confounders, p∈2,4,6,10, and (iii) the effect size, with β=(0.5,0) (small), β=(0.7,0) (moderate), β=(0.9,0) (moderately large), and β=(1.0,0) (large) for the single effect modifier. We conduct 1000 simulation replicates for each setting. The observed patterns are consistent with those reported in the main simulation study (Section 4).

### Simulation Results Under Additional Effect Size Scenarios

In the main text, we present results for small (β=(0.6,0,0,0.3)) and large (β=(1.2,0,0,0.4)) treatment effect sizes. Here, we additionally include results for moderate (β=(0.8,0,0,0.4)) and moderately large (β=(0.9,0,0,0.3)) effect sizes. Results for hypothesis testing and effect modifier identification are shown in Figures D3 and D4, respectively. The patterns are consistent with those reported in the simulation section (Section 4). Overall, the performance of each method improves as effect size increases, with HSR showing the most pronounced change and submax+ exhibiting a more stable performance across effect sizes.

### Statistical Power for Testing the Global Null Hypothesis

We report statistical power for testing the global null hypothesis of no treatment effect across all matched sets. For HSR, the global null hypothesis is rejected if its p‐value, computed as the truncated product of the p‐values for testing the subgroup null hypothesis $H_{g}$ for g=1,…,G (Section 3.1), is at most α=0.05. Here, G denotes the number of subgroups defined by leaf nodes in the CART tree. For submax and related methods, the global null hypothesis is rejected if $D_{\Gamma max}=\max_{1\leq k\leq K}D_{\Gamma k}$ exceeds the critical constant at level α=0.05 described in Section 3.2. submax (best) always uses K=1, while submax use K=2p+1, respectively, where p is the number of confounders. submax+ uses K=2L+1, where L variables are selected for splitting in the CART tree.

Figure D5 displays the TPR (empirical power) across 1000 replicates for the simulation settings in Section 4. Power increased with effect size and with the number of matched pairs I, and declined modestly as the number of confounders p grew. submax and submax+ performed very similarly, indicating that the adaptive choice of L had little impact on power for testing the global null. HSR was more sensitive to sample size: at smaller I (e.g., I=200), it showed lower power than submax or submax+ , but for large effect sizes and sufficiently large I (e.g., I≥400), its power exceeded others. When the effect size was small to moderate, all three methods had comparable power.
