# Supplementary material for: Comparison of Methods for Sensitivity Analysis of Heterogeneous Treatment Effects in Observational Studies and Application to Alzheimer's Disease and Cognitive Decline
Source: Stat Med. 2026 Mar 17;45(6-7):e70446. doi: 10.1002/sim.70446 (PMC12995544; doi:10.1002/sim.70446)

How to cite this article: Jingqi Duan, Corinne D. Engelman, Qiongshi Lu, and Hyunseung Kang. Comparison of Methods for Sensitivity Analysis of Heterogeneous Treatment Effects in Observational Studies and Application to Alzheimer's Disease and Cognitive Decline. 2000;00(00):1–18.

APPENDIX

A TRIMMED, WEIGHTED M-STATISTICS

The weighted M-statistic with inner trimming, an extension of Maritz's version[41] of Huber's[42] M-statistic, is proposed by Rosenbaum[43,44,45] to increase the power of a sensitivity analysis. Under Fisher's sharp null of no effect, $R_{ij} = r_{Cij}$, $R_{ij} - R_{il} = r_{Cij} - r_{Cil}$, and the weighted M-statistic with inner trimming becomes

$$\begin{aligned} T_g &= \sum_{i \in s_g} w_i \sum_{j=1}^{n_i} Z_{ij} \sum_{l=1}^{n_i} \psi_{\text{in}}\{(r_{Cij} - r_{Cil})/s\} \\ &= \sum_{i \in s_g} w_i \sum_{j=1}^{n_i} Z_{ij} q_{ij}, \;\; q_{ij} = \sum_{l=1}^{n_i} \psi_{\text{in}}\{(r_{Cij} - r_{Cil})/s\}, \end{aligned} \tag{A1}$$

where

- w_i is non-negative with $w_i \propto \tilde{w}_i = \sum_{l=\underline{m}}^{\overline{m}} \binom{a_i-1}{l-1}\binom{I_g-a_i}{m-l}$, where $(m, \underline{m}, \overline{m})$ are weights with $1 \le \underline{m} \le \overline{m} \le m$ and a_i is the rank of the ranges $\max_{1\le j\le n_i} q_{ij} - \min_{1\le l\le n_i} q_{il}$ with q_{ij} defined in (A1); refer to Section 4.2 in Rosenbaum[44] for details.
- $\psi_{\text{in}}(y) = \{h/(h-\iota)\} \cdot \text{sign}(y) \cdot \max\{0, \ \min(|y|, \ h) - \iota\}$, where the terms h and ι are outer and inner trimming with $0 \le \iota < h$ and $\psi_{\text{in}}(0) = 0$; see Section 6.2 in Rosenbaum[45] for details.
- s is the λ quantile of all pairwise absolute differences $|R_{ij} - R_{il}|$, $1 \le j < l \le n_i$, $i \in s_g$ with $0 < \lambda < 1$. For example, $\lambda = 1/2$ yields the median of the absolute difference and s is the value of the median.

B LITERATURE REVIEW OF SLEEP PROBLEMS

Sleep problems have been defined in various ways in the literature, including, but not limited to, reduced slow-wave sleep and rapid eye movement sleep[11], sleep fragmentation[9], sleep-disordered breathing[5], and both short[2] and long sleep duration[4]. The definitions of sleep problems often depend on the specific phenomena that the investigator aims to study.

Sleep quality is often considered a symptom or marker of sleep problems. Although it is an important and modifiable factor in sleep research, its definition also varies across studies[56,57,58]. For example, a recent meta-analysis of 65 randomized controlled trials[59] examined the causal effects of sleep-improving interventions on mental health outcomes. In this study, sleep quality was assessed based on sleep continuity and daytime functioning, but as the authors note, "...different sleep disorders have different conceptualisations of improvement that might not include sleep quality."

In our study, the definition of sleep problems was based on data availability and a recent recommendation[58], which suggested using self-reported assessments from different perspectives, such as self-reported and caregiver-reported sleep issues. Section 6 further discusses how to interpret our results within the broader context of other research on sleep problems and cognitive decline.

C FURTHER DETAILS OF DATA ANALYSIS

C.1 Processing of Genetic Data

To adjust for potential genetic confounding, we constructed polygenic risk scores (PRS) for insomnia, chronotype, and sleep duration. Specifically, we followed the procedure outlined in Zhao et al.[60] and used PRSice-2[61] to compute the PRS. The genome-wide association study (GWAS) summary statistics used to generate the PRS were first pruned using PLINK[62]. We set the linkage disequilibrium (LD) block window to 100 variants, with a step size of 5 variants to shift the window, and applied a pairwise LD (r^2) threshold of 0.1. LD estimation was based on European-ancestry samples from the 1000 Genomes Project Phase III, which served as the reference panel.

C.2 Study Population

Table C1 summarizes covariates, exposure, and outcome between the analysis sample ($n = 1131$) and the original sample ($n = 2430$). Overall, most covariates between the analysis sample and the original sample are similar except five covariates. Specifically, the proportion of females is slightly lower in the analysis sample (43. 8%) than in the original sample (46.8%). The proportion of individuals identifying as Hispanic or Latino is slightly lower in the analysis sample (1.3%) than in the original sample

(3.6%). The analysis sample has fewer missing values for family history than the original sample. The analysis sample shows higher baseline composite memory score (0.36) and executive function score (0.30) than those in the original sample (0.23 and 0.15, respectively). But the outcome, which is defined as the change from baseline measurement, is similar in both samples. We remark that the analysis sample does not have missing values for genetic data (polygenic risk scores and APOE gene), which is expected since our analysis sample only considers individuals who have their genetic data measured.

The prevalence of the exposure, sleep problems, is slightly lower in the analysis sample (26.1%) compared to the original sample (29.8%). Note that the exposure and outcome variables have a large proportion of missing data in the original sample, which is expected since our analysis sample only considered individuals that had their exposure and outcome measured.

TABLE C1 Summary statistics of 31 covariates at baseline.

	Analysis Sample (n=1131)		Original Sample (n=2430)	
Covariates	Mean (SD)	%Missing	Mean (SD)	%Missing
Sex (Female)	43.8%		46.6%	0.5%
Latino ethnicity	1.3%		3.6%	0.5%
Age at baseline, years	73.68 (7.04)		73.00 (7.54)	0.5%
Retirement age, years	74.10 (23.91)	1.5%	74.65 (24.58)	2.5%
Education, years	16.07 (2.76)		15.98 (2.82)	0.5%
Right-handed	91.4%		91.2%	0.5%
Residence type				
House	77.3%		75.2%	0.5%
Condo/co-op	12.3%		12.1%	0.5%
Rented apartment	5.0%		7.7%	0.5%
Retirement community	3.0%		2.7%	0.5%
Mobile home	1.1%		0.9%	0.5%
Assisted living	0.4%		0.5%	0.5%
Marital status				
Married	77.6%		74.4%	0.5%
Widowed	10.9%		11.1%	0.5%
Divorced	8.0%		9.7%	0.5%
Never married	3.1%		4.2%	0.5%
Family history				
Mom with dementia	40.6%	2.3%	40.6%	8.6%
Mom with AD	26.9%	8.7%	25.9%	16.3%
Dad with dementia	18.4%	4.6%	18.3%	10.8%
Dad with AD	10.1%	8.0%	9.7%	14.4%
Number of female siblings	1.17 (1.27)	0.6%	1.22 (1.32)	6.4%
Number of siblings w/o dementia and AD	2.05 (1.99)	0.6%	2.15 (2.04)	6.4%
Number of siblings with dementia	0.06 (0.28)	0.6%	0.05 (0.26)	6.4%
Number of siblings with AD	0.15 (0.46)	0.6%	0.13 (0.45)	6.4%
APOE $\varepsilon 2$ copies (0/1/2)	90.5/9.5/0.0%		91.1/8.8/0.1%	37.9%
APOE $\varepsilon 4$ copies (0/1/2)	54.4/36.1/9.5%		52.6/37.5/9.9%	37.9%
Polygenic risk scores ($\times 10^{-6}$)				
Insomnia	-6.08 (20.01)		-5.99 (19.63)	37.9%
Sleep duration	84.58 (8.45)		84.52 (8.35)	37.9%
Chronotype	-39.87 (8.41)		-39.85 (8.45)	37.9%
Baseline composite memory score	0.34 (0.86)		0.23 (0.90)	32.1%
Baseline composite executive function score	0.31 (1.00)		0.15 (1.04)	32.1%
Exposure: Sleep problems	26.1%		29.8%	25.6%
Outcome: Decline in composite executive function score	0.10 (0.69)		0.09 (0.69)	47.2%

D FURTHER DETAILS OF SIMULATION STUDY

D.1 Simulation Results with a Single Effect Modifier

In the main text, we present results for scenarios with two binary effect modifiers. In addition, we conducted a simulation study with a single binary effect modifier under similar settings. Specifically, $G = 2$ subgroups are defined by $x_1 = 0$ and $x_1 = 1$. The results for hypothesis testing and effect modifier identification are shown in Figures D3 and D4, respectively. Specifically, we vary (i) the number of matched pairs, $I \in 200, 400, 600$, (ii) the number of confounders, $p \in 2, 4, 6, 10$, and (iii) the effect size, with $\boldsymbol{\beta} = (0.5, 0)$ (small), $\boldsymbol{\beta} = (0.7, 0)$ (moderate), $\boldsymbol{\beta} = (0.9, 0)$ (moderately large), and $\boldsymbol{\beta} = (1.0, 0)$ (large) for the single effect

modifier. We conduct 1,000 simulation replicates for each setting. The observed patterns are consistent with those reported in the main simulation study (Section 4).

FIGURE D1 F_1 score and true positive rate (TPR) for hypothesis testing under different simulation settings with one binary effect modifier. Columns correspond to treatment effect sizes (small: $\boldsymbol{\beta} = (0.5, 0)$, moderate: $\boldsymbol{\beta} = (0.7, 0)$, moderately large: $\boldsymbol{\beta} = (0.9, 0)$, and large: $\boldsymbol{\beta} = (1.0, 0)$) and the number of matched pairs (I). Rows correspond to the number of confounders (p). Sensitivity parameter values (Γ) range from 1 to 6 in increments of 0.2.

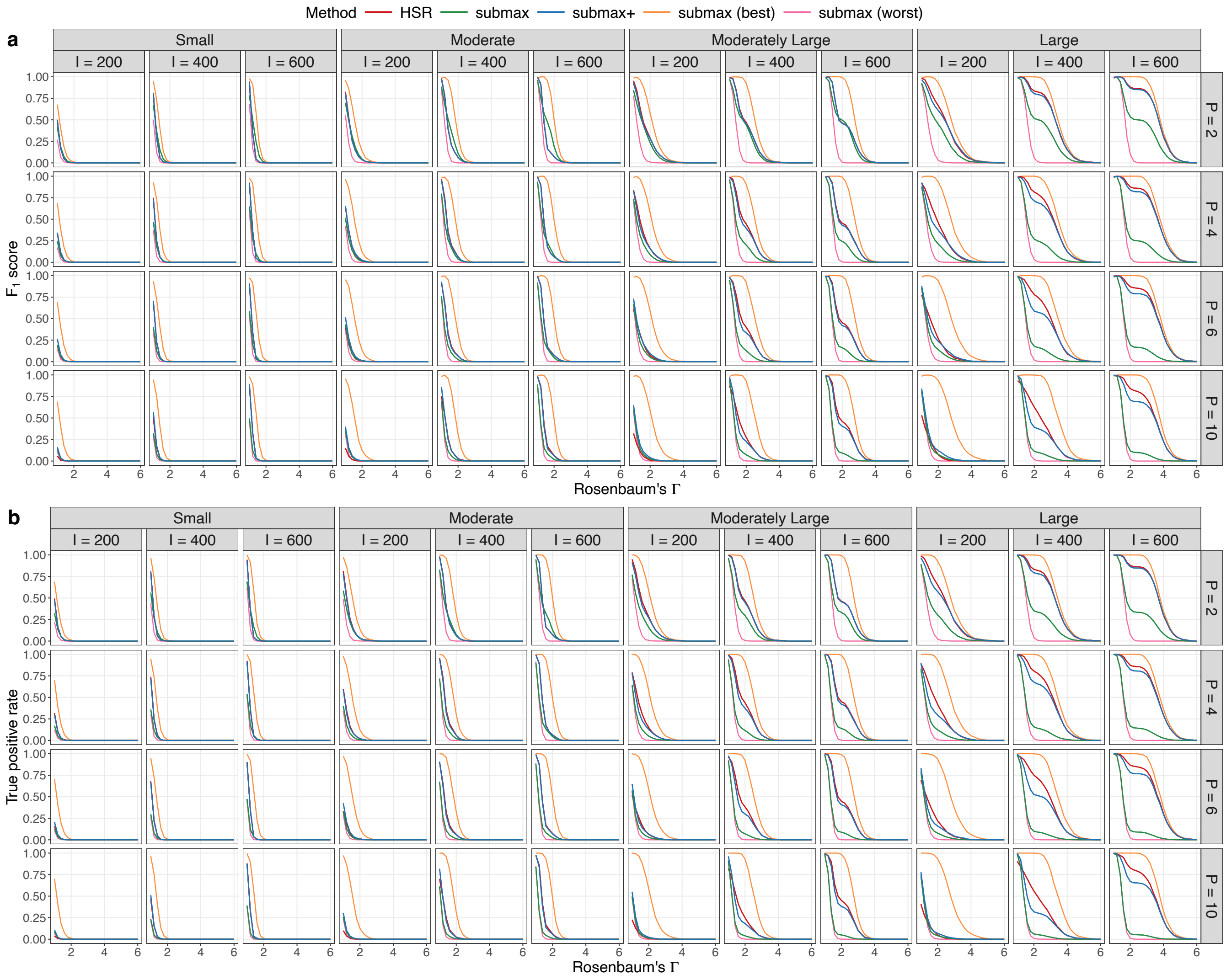

D.2 Simulation Results under Additional Effect Size Scenarios

In the main text, we present results for small ($\boldsymbol{\beta} = (0.6, 0, 0, 0.3)$) and large ($\boldsymbol{\beta} = (1.2, 0, 0, 0.4)$) treatment effect sizes. Here, we additionally include results for moderate ($\boldsymbol{\beta} = (0.8, 0, 0, 0.4)$) and moderately large ($\boldsymbol{\beta} = (0.9, 0, 0, 0.3)$) effect sizes. Results for hypothesis testing and effect modifier identification are shown in Figures D3 and D4, respectively. The patterns are consistent with those reported in the simulation section (Section 4). Overall, the performance of each method improves as effect size increases, with `HSR` showing the most pronounced change and `submax+` exhibiting a more stable performance across effect sizes.

D.3 Statistical Power for Testing the Global Null Hypothesis

We report statistical power for testing the global null hypothesis of no treatment effect across all matched sets. For `HSR`, the global null hypothesis is rejected if its p-value, computed as the truncated product of the p-values for testing the subgroup null hypothesis H_g for $g = 1, \ldots, G$ (Section 3.1), is at most $\alpha = 0.05$. Here, G denotes the number of subgroups defined by leaf

FIGURE D2 F_1 score and true positive rate (TPR) for effect modifier identification under different simulation settings with one binary effect modifier. Columns correspond to treatment effect sizes (small: $\boldsymbol{\beta} = (0.5, 0)$, moderate: $\boldsymbol{\beta} = (0.7, 0)$, moderately large: $\boldsymbol{\beta} = (0.9, 0)$, and large: $\boldsymbol{\beta} = (1.0, 0)$) and the number of matched pairs (I). Rows correspond to the number of confounders (p). Sensitivity parameter values (Γ) range from 1 to 6 in increments of 0.2.

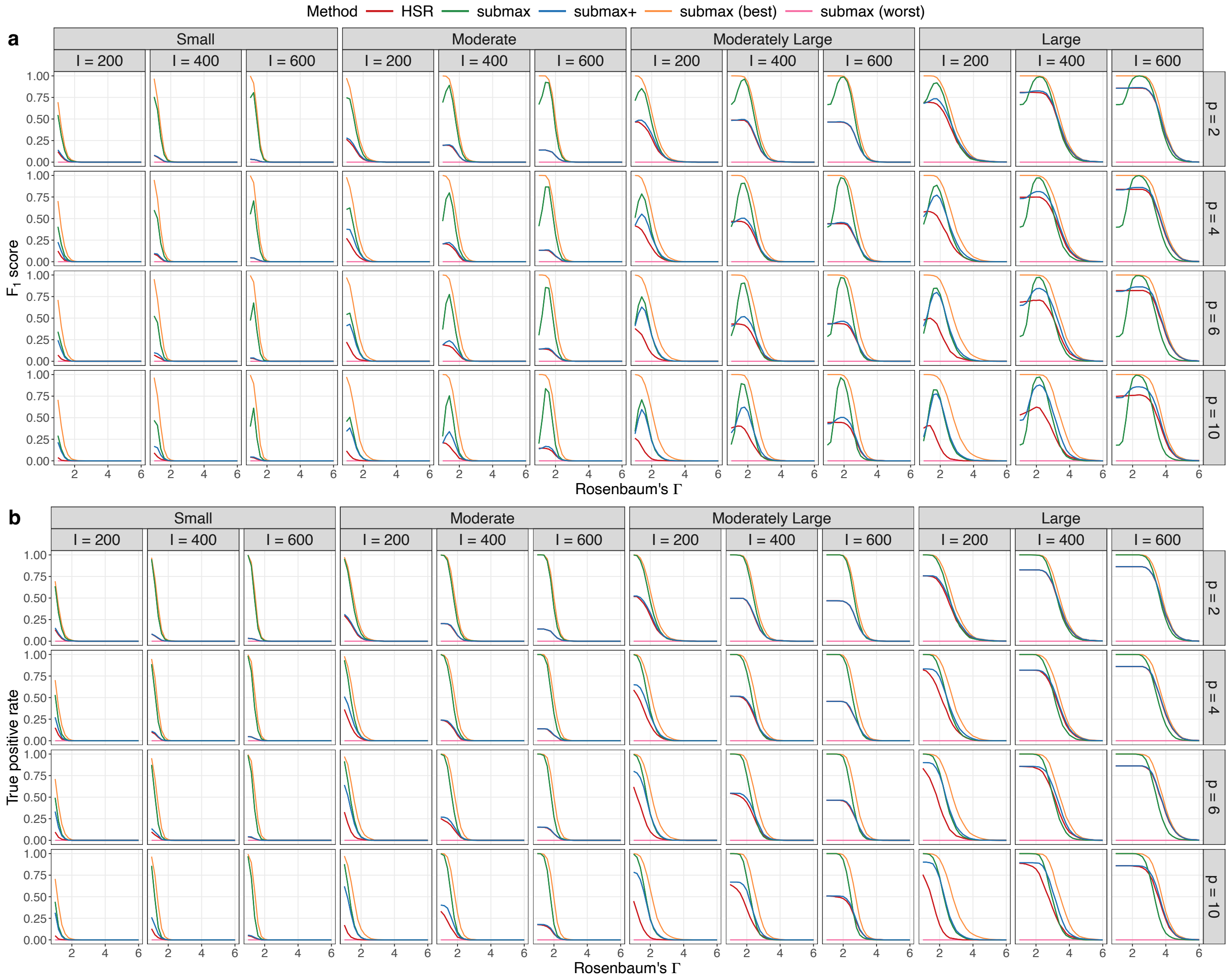

nodes in the CART tree. For `submax` and related methods, the global null hypothesis is rejected if $D_{\Gamma max} = \max_{1 \le k \le K} D_{\Gamma k}$ exceeds the critical constant at level $\alpha = 0.05$ described in Section 3.2. `submax (best)` always uses $K = 1$, while `submax` use $K = 2p + 1$, respectively, where p is the number of confounders. `submax`+ uses $K = 2L + 1$, where L variables are selected for splitting in the CART tree.

Figure D5 displays the true positive rate (empirical power) across 1,000 replicates for the simulation settings in Section 4. Power increased with effect size and with the number of matched pairs I, and declined modestly as the number of confounders p grew. `submax` and `submax`+ performed very similarly, indicating that the adaptive choice of L had little impact on power for testing the global null. `HSR` was more sensitive to sample size: at smaller I (e.g., $I = 200$), it showed lower power than `submax` or `submax`+, but for large effect sizes and sufficiently large I (e.g., $I \geq 400$), its power exceeded others. When the effect size was small to moderate, all three methods had comparable power.

FIGURE D3 F_1 score and true positive rate (TPR) for hypothesis testing under different simulation settings with two binary effect modifier. Columns correspond to treatment effect sizes (small: $\boldsymbol{\beta} = (0.6, 0, 0, 0.3)$, moderate: $\boldsymbol{\beta} = (0.8, 0, 0, 0.4)$, moderately large: $\boldsymbol{\beta} = (0.9, 0, 0, 0.3)$, and large: $\boldsymbol{\beta} = (1.2, 0, 0, 0.4)$) and the number of matched pairs (I). Rows correspond to the number of confounders (p). Sensitivity parameter values (Γ) range from 1 to 6 in increments of 0.2.

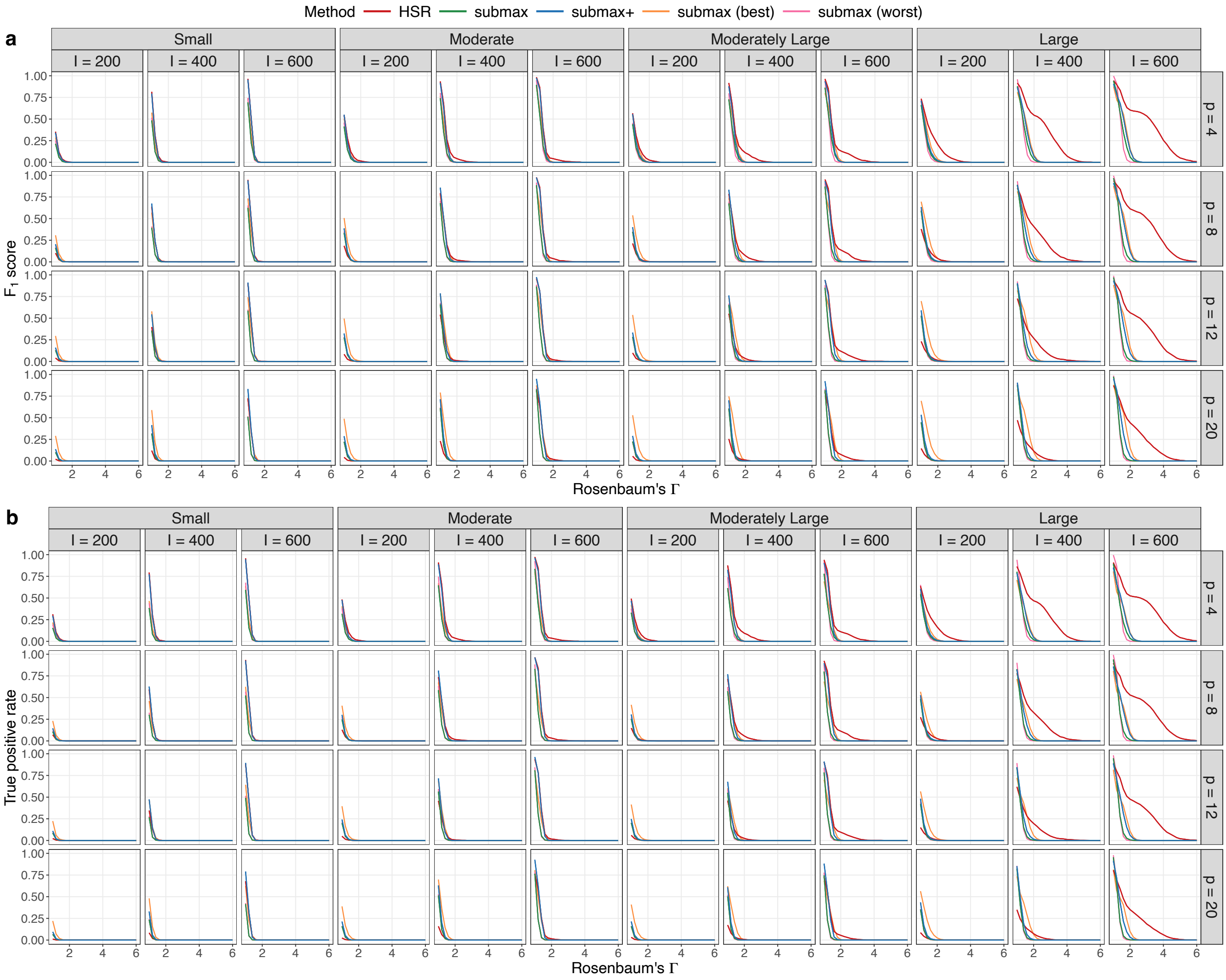

FIGURE D4 F_1 score and true positive rate (TPR) for effect modifier identification under different simulation settings with two binary effect modifiers. Columns correspond to treatment effect sizes (small: $\boldsymbol{\beta} = (0.6, 0, 0, 0.3)$, moderate: $\boldsymbol{\beta} = (0.8, 0, 0, 0.4)$, moderately large: $\boldsymbol{\beta} = (0.9, 0, 0, 0.3)$, and large: $\boldsymbol{\beta} = (1.2, 0, 0, 0.4)$) and the number of matched pairs (*I*). Rows correspond to the number of confounders (*p*). Sensitivity parameter values (Γ) range from 1 to 6 in increments of 0.2.

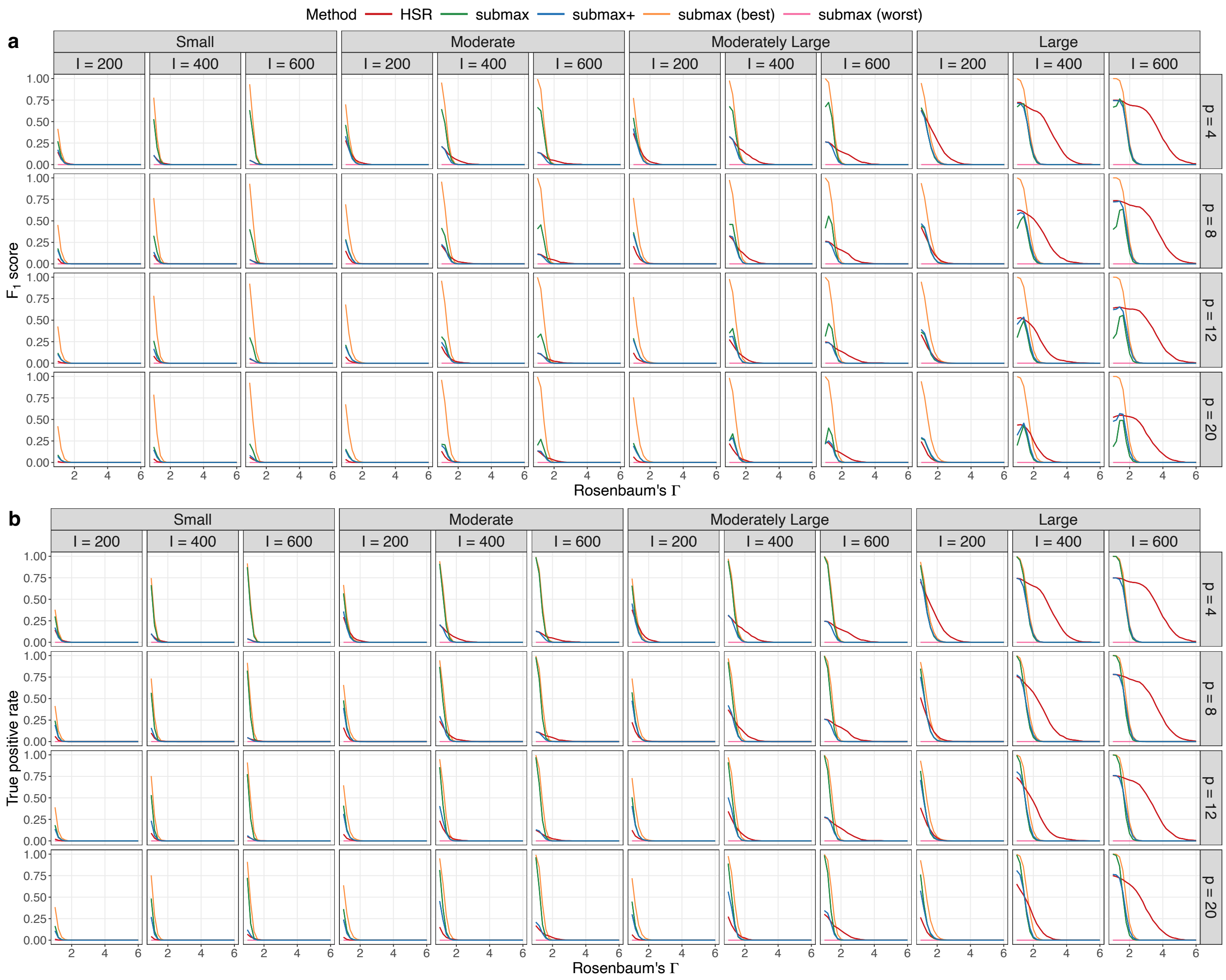

FIGURE D5 True positive rate (TPR) for testing global null hypothesis under different simulation settings. Columns correspond to treatment effect sizes (small: $\boldsymbol{\beta} = (0.6, 0, 0, 0.3)$, moderate: $\boldsymbol{\beta} = (0.8, 0, 0, 0.4)$, moderately large: $\boldsymbol{\beta} = (0.9, 0, 0, 0.3)$, and large: $\boldsymbol{\beta} = (1.2, 0, 0, 0.4)$) and the number of matched pairs (*I*). Rows correspond to the number of confounders (*p*). Sensitivity parameter values (Γ) range from 1 to 6 in increments of 0.2.

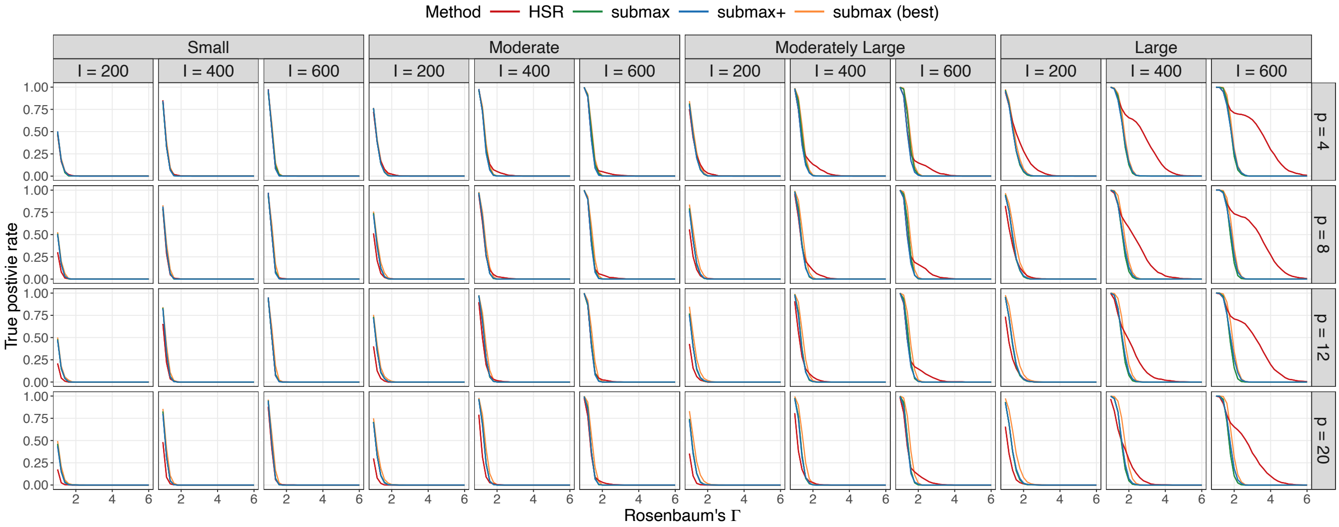

Supplement: Supplementary file 1 — Data S1: Additional supporting information may be found in the online version of the article at the publisher's website. [file SIM-45-0-s001.pdf]
